# RNA modifications in health and disease: from mechanistic insights to therapeutic applications

**DOI:** 10.1093/pcmedi/pbaf035

**Published:** 2025-11-26

**Authors:** Yiting Chen, Dulin Ding, Xing Tang, Rui Ma, Jian-Kang Zhou

**Affiliations:** Department of Pathology and Pathophysiology, School of Basic Medical Sciences, Chengdu Medical College, Chengdu 610500, China; Department of Pathology and Pathophysiology, School of Basic Medical Sciences, Chengdu Medical College, Chengdu 610500, China; School of Clinical Medicine, Chengdu Medical College, Chengdu 610500, China; Department of Hematology & Hematopoietic Cell Transplantation, City of Hope National Medical Center, Los Angeles, CA 91010, USA; Hematologic Malignancies Research Institute, City of Hope National Medical Center, Los Angeles, CA 91010, USA; Department of Pathology and Pathophysiology, School of Basic Medical Sciences, Chengdu Medical College, Chengdu 610500, China; Institute of Geriatric Cardiovascular Disease, Chengdu Medical College, Chengdu 610500, China

**Keywords:** RNA modification, methylation, m^6^A, pseudouridine, ac^4^C, RNA editing

## Abstract

RNA modifications encompass a series of dynamic chemical changes and editing events on RNA molecules, playing a pivotal role in essential physiological processes such as embryonic development, immune response, and the maintenance of cell homeostasis. By influencing RNA stability, splicing, translation, and intermolecular interactions, RNA modifications serve as crucial mechanisms regulating gene expression at the post-transcriptional level. Dysregulation of the modification machineries or aberrant modification patterns is closely associated with the onset and progression of various diseases, including tumors, metabolic disorders, cardiovascular diseases, and neurological and immune conditions, making them potential biomarkers for disease diagnosis, prognosis, and treatment. In this review, we summarize the molecular mechanisms of major RNA modifications, emphasize their functions in health and disease, and discuss their diagnostic and therapeutic value in pathological contexts.

## Introduction

RNA modifications are commonly found in various RNA types, including messenger RNA (mRNA), transfer RNA (tRNA), ribosomal RNA (rRNA), and small nuclear RNA (snRNA), with mRNA modifications being the most extensively researched [1–3]. In 1951, scientists first discovered pseudouridine (Ψ) in yeast RNA, marking the beginning of RNA modification research [4]. Subsequently, modifications such as *N*^6^-methyladenosine (m^6^A) (1955) [5], 5-methylcytidine (m^5^C) (1958) [6], and m^1^A(1961) [7] were successively identified. Recently, a new type of glycosylated RNA (glycoRNA) was discovered, challenging the traditional understanding that glycosylation occurs exclusively in proteins or lipids [8, 9]. To date, over 170 distinct RNA modifications have been identified [10], many of which are highly conserved across bacteria, archaea, and eukaryotes, while others are specific to certain organism classes [11, 12]. These modifications significantly enhance the functional diversity of RNA by regulating its structure, stability, splicing, translation efficiency, and interactions with other molecules [13, 14].

**Figure 1.**
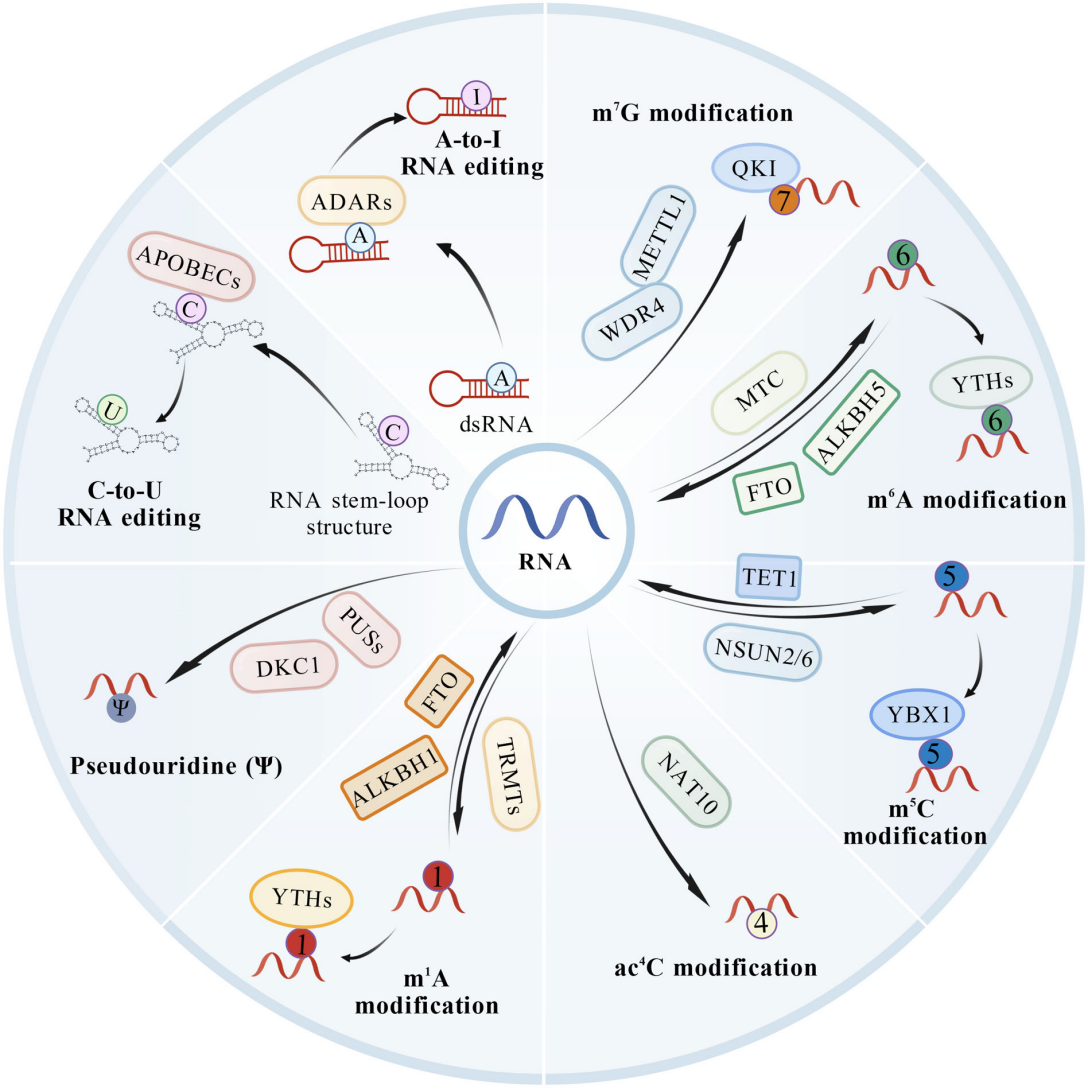
Common types of RNA modifications. RNA molecules, including messenger RNA (mRNA), transfer RNA (tRNA), and ribosomal RNA (rRNA), undergo various modifications through the addition of specific chemical groups. Additionally, RNA editing is also a common form of modification, such as adenosine-to-inosine (A-to-I) RNA editing.

The most extensively studied types of RNA modifications include m^6^A, *N*^1^-methyladenosine (m^1^A), m^5^C, *N*^6^,*2’*-*O*-dimethyladenosine (m^6^Am), *N*^7^-methylguanosine (m^7^G), Ψ, ac^4^C, and the editing of adenosine-to-inosine (A-to-I) and cytidine-to-uridine (C-to-U). Each modification possesses core regulatory factors and specific modification sites [15] (Fig. 1). Given that RNA modifications are key factors in regulating gene expression, responding to environmental stress, transducing signal pathways, determining cell fate, enabling immune recognition, and controlling protein synthesis, they play a central role in numerous biological processes [16–18]. More importantly, owing to their dynamic and reversible nature, RNA modifications exert indispensable functions in core life processes such as embryonic development, stem cell fate determination, and tissue differentiation [19, 20].

RNA modifications are dynamically regulated by specific enzyme systems, which are typically categorized into writer and eraser [21, 22]. Specifically, writers are responsible for adding modification groups to RNA molecules, while erasers remove these modification groups from RNA, ensuring the reversibility of the modification process. This enzyme system confers significant plasticity to RNA modifications, enabling rapid responses to intracellular changes and external regulation [15, 23]. Modified RNA molecules are further recognized and bound by the reader proteins, thereby regulating their fate and function [24, 25]. Dysregulation of these modification machineries, whether arising from genetic mutations, epigenetic changes, or environmental disturbances, is increasingly linked to diverse diseases, underscoring their significance as potential therapeutic targets [26]. Additionally, abnormal RNA modifications, such as alterations in the type, location, or extent of chemical modifications on RNA molecules, can also affect RNA function or stability. This may subsequently contribute to the occurrence and development of various diseases, including cancer, cardiovascular diseases, nervous system disorders, and metabolic diseases [20, 27, 28].

In this review, we systematically summarize chemical modifications that do not alter base characteristics (such as m^6^A) and RNA editing that changes genetic information (such as A-to-I RNA editing), describe their features, regulatory mechanisms, and functions in health and disease. We also discuss potential therapeutic strategies and prospects targeting these modifications and their regulatory machineries.

## m^6^A

m^6^A is the most abundant internal methylation modification in mRNA and long non-coding RNA (lncRNA), characterized by the addition of a methyl group (-CH3) to the sixth nitrogen atom of adenosine (A) [29, 30]. Typical m^6^A modification sites exhibit DRACH sequence (D = G, A or U; R = G or A; H = A, C or U), and are enriched in coding sequences (CDS) and 3’-untranslated regions (3’-UTR), particularly concentrated in the area of the termination codon [31]. As a crucial epigenetic regulator, m^6^A plays significant roles in diverse biological processes.

### Dynamic regulation of m^6^A modification

The dynamic regulation of m^6^A modification relies on the precise collaboration between writers and erasers, which jointly shape the m^6^A modification landscape on RNA molecules.

m^6^A deposition is mainly dependent on the methyltransferase complex (MTC). This complex features a core catalytic unit comprising the methyltransferase-like 3 (METTL3) and METTL14 heterodimer, supported by regulatory factors such as Wilms’ tumor 1-associating protein (WTAP), Vir-like m^6^A methyltransferase-associated (VIRMA, also known as KIAA1429), RNA-binding motif protein 15 (RBM15), and zinc finger CCCH-type containing 13 (ZC3H13) [32, 33]. METTL3 performs the catalytic function by transferring methyl groups to RNA through binding to the methyl donor S-adenosylmethionine (SAM) [34]. However, METTL14 exhibits minimal catalytic activity despite containing a catalytic domain, primarily contributing to maintaining METTL3 structural stability and guiding the MTC to specific mRNAs [35, 36]. Other components, such as WTAP, enhance MTC function by optimizing complex assembly and substrate selectivity [37].

In addition to MTC, several independent methyltransferases are responsible for m^6^A modification of specific RNA molecules. For instance, METTL16 catalyzes the m^6^A modification on U6 snRNA by recognizing the UACAGAGAA sequence. Moreover, METTL16 negatively regulates SAM levels through feedback control of m^6^A modification on MAT2A mRNA [38, 39]. ZCCHC4 has been identified as the writer responsible for the 28S rRNA m^6^A modification. Knockdown of ZCCHC4 eliminates m^6^A modification in 28S rRNA, leading to reduced overall translation and inhibition of cell proliferation [40]. METTL5 also functions as a methyltransferase, forming a complex with TRMT112 to catalyze m^6^A methylation of 18S rRNA [41]. Deletion of METTL5 affects gene expression at the translation level and results in metabolic defects [41].

RNA modification can be reversed by the demethylase (eraser), enabling reversible m^6^A regulation. Currently, the main m^6^A erasers are fat mass and obesity-associated protein (FTO) and AlkB homolog 5 (ALKBH5), both of which require Fe^2+^ as a cofactor and α-ketoglutaric acid as a substrate to achieve m^6^A demethylation through oxidation [42–44]. As the first identified m^6^A eraser, FTO primarily regulates m^6^A modifications by removing the methyl group from specific transcripts [45]. Besides m^6^A, FTO can also mediate the demethylation of m^6^Am and m^1^A modifications. In contrast, ALKBH5 functions exclusively as an eraser for mRNA m^6^A [46]. The expression of ALKBH5 directly influences m^6^A levels of target RNAs, thereby regulating mRNA export, RNA metabolism, and the assembly of mRNA processing factors in nuclear speckles [44].

### Modulatory effects of m^6^A modification

**Figure 2.**
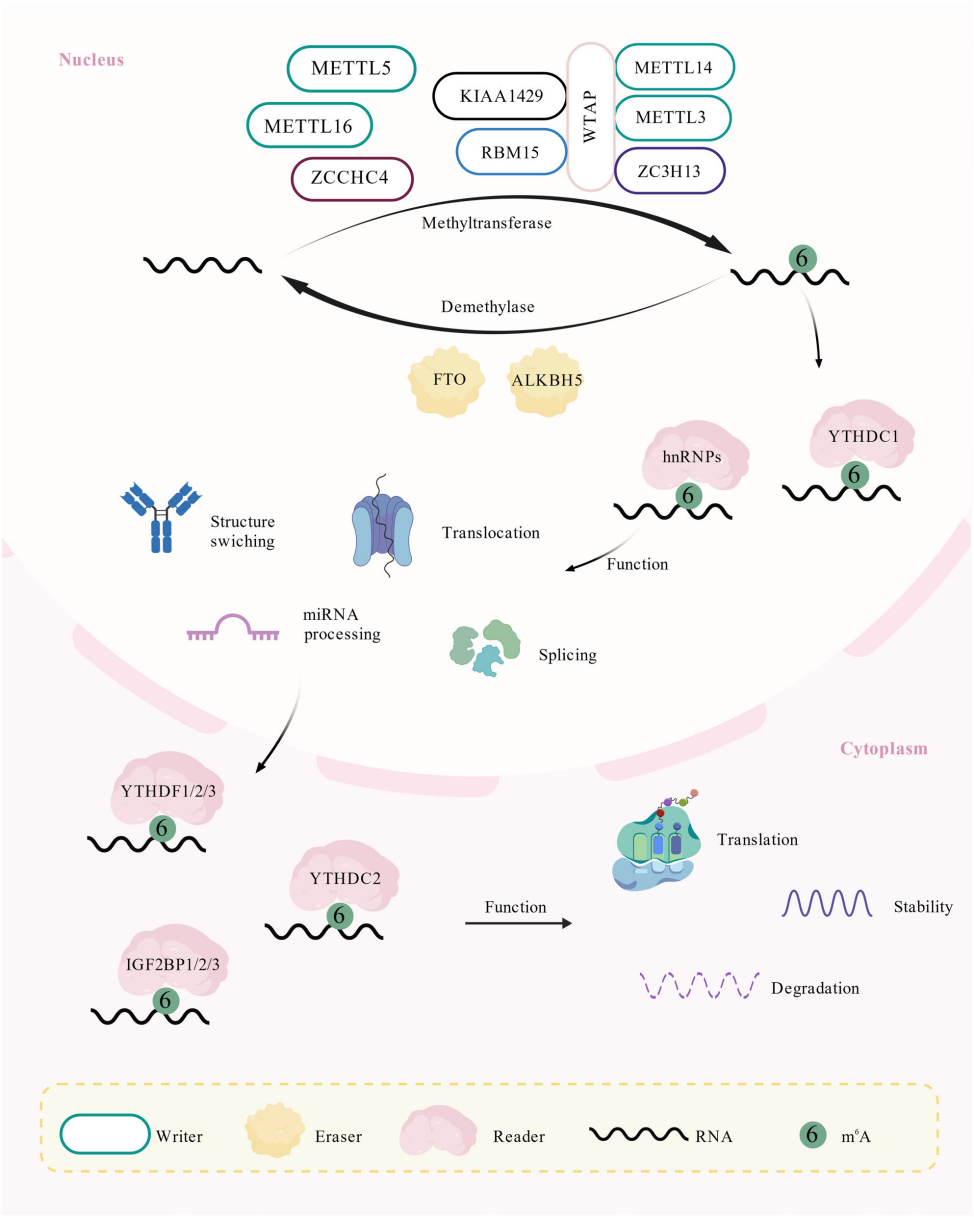
The dynamic regulation and functional roles of m^6^A RNA modification. The *N*^6^-methyladenosine (m^6^A) modification is dynamically and reversibly regulated by writers and erasers. Writer complexes, primarily composed of METTL3/METTL14, catalyze the deposition of m^6^A marks on specific RNA transcripts. Conversely, eraser proteins like FTO and ALKBH5 remove these methyl groups, ensuring a reversible process. This dynamic modification directly influences RNA fate by recruiting distinct reader proteins. These readers, such as the YTHDF family, recognize and bind to m^6^A sites. Depending on the reader and cellular context, this binding triggers diverse downstream consequences, including alterations in RNA splicing, export, translation efficiency, stability, and degradation.

Following m^6^A modification, a class of proteins specifically recognizes and binds the modified site, thereby regulating mRNA splicing, translation, degradation, stability, and nucleoplasmic transport (Fig. 2). These proteins, known as readers, exhibit diverse structures and functions, responsible for translating m^6^A markers into corresponding functional effects.

The first category is the YTH domain protein family, the most extensively studied m^6^A reader, whose members are distributed in both the cytoplasm and nucleus [47]. In the cytoplasm, YTH domain family 1 (YTHDF1) enhances translation by binding to the eukaryotic initiation factor 3 (eIF3) and increasing the loading efficiency of ribosomes on m^6^A-modified mRNAs [48]. YTHDF2 mediates the degradation of target mRNA by recruiting the CCR4-NOT deaminase complex [49, 50], while YTHDF3 synergistically promotes translation with YTHDF1 and collaborates with YTHDF2 to accelerate mRNA degradation [51]. In contrast, YTHDC1 primarily localizes to the nucleus, participating in splicing of m^6^A-modified precursor mRNAs and nuclear transport of mature mRNAs [52–54]. Insulin-like growth factor 2 mRNA binding proteins (IGF2BP1/2/3) also function as m^6^A readers. They selectively bind m^6^A-modified RNA through their KH3 and KH4 domains, recruiting RNA stabilization factors, such as ELAV-like RNA binding protein 1 (ELAVL1) or translation initiation factors (eIFs), thereby enhancing the stability and translation efficiency of target mRNA [55]. In addition, they competitively occupy m^6^A sites on target transcripts, interfering with miRNA-mediated mRNA degradation and thus maintaining mRNA levels [56]. Another class of m^6^A readers comprises members of the heterogeneous nuclear ribonucleoprotein (hnRNP) family, such as hnRNP A2B1, hnRNP G, and hnRNP C, which can directly recognize m^6^A-modified pri-miRNAs or lncRNAs [57]. By interacting with m^6^A, these proteins can alter the local secondary structure of RNA, thereby regulating the maturation of pri-miRNA and the interaction between lncRNA and proteins [57, 58]. Besides, the recently identified m^6^A RNA-binding protein RBM33 adds complexity to m^6^A regulation. It recognizes and binds m^6^A-modified RNA substrates through its RNA recognition domain (RRM), recruiting ALKBH5 and the small ubiquitin-like modifier (SUMO) ligase SENP1 [59]. SENP1 further activates demethylase activity by inhibiting the SUMOylation of ALKBH5, thereby promoting m^6^A demethylation in specific RNA transcripts [59]. This mechanism reveals a novel regulatory module that precisely modulates m^6^A levels in a transcript-specific manner.

m^6^A-modified RNA, upon recognition by readers, influences gene expression and cellular functions by regulating RNA metabolism, transcription, and translation processes. For instance, m^6^A modification promotes the splicing and maturation of pri-miRNAs, which is essential for the accurate generation of miRNAs that mediate post-transcriptional gene silencing [60, 61]. For lncRNAs, theire functions are heavily influenced by m^6^A modification, which regulates alternative splicing in the nucleus, acts as a competing endogenous RNA (ceRNA) in the cytoplasm, and contributes to transcriptional activation through enhancer RNA (eRNA) [62]. Additionally, some lncRNAs can inversely regulate the m^6^A modification mechanism, playing a role in the onset and progression of diseases [62]. Furthermore, the regulatory scope of m^6^A extends to circular RNAs (circRNAs). m^6^A modification promotes circRNA translation by recruiting YTHDF3 and eukaryotic initiation factor eIF4G2, challenging the conventional notion that “circRNA cannot encode proteins” [63].

In addition to directly affecting RNA function, the expression of m^6^A-related enzymes is precisely regulated by multiple intracellular and extracellular signals, forming a dynamic feedback loop between cells and their environment. Under hypoxic conditions, HIF-1α regulates m^6^A modification through a dual mechanism to adapt to the environment. On one hand, it promotes WTAP expression and stimulates aerobic glycolysis in tumor cells via the miRNA-200-hexokinase 2 (HK2) axis [64]. On the other hand, it inhibits METTL14 expression, thereby preventing YTHDF2-mediated degradation of *SLC7A11* mRNA, helping to balance cellular oxidative stress [65]. Interestingly, even gut microbiota metabolites, such as butyrate, can alter the m^6^A methylation level of RNA, highlighting the unexpected role of m^6^A in host-microbe interactions [66].

Therefore, m^6^A modification and its recognition system exert significant biological influence across basic RNA metabolism to complex physiological processes, basedon their precise regulation of RNA fate.

### The role of m^6^A in diseases

The dynamics and reversibility of m^6^A modification are crucial for maintaining normal cellular functions. Disruption of m^6^A regulation impacts gene expression and contributes to the onset and progression of various diseases. The key role of m^6^A modification in tumors (Table 1), metabolic diseases, cardiovascular diseases, immunity and inflammation, and nervous system diseases (Table 2) highlights its significant potential as an important pathological factor and therapeutic target.

**Table 1.** The role of RNA m^6^A modification-related regulatory factors in tumors.

Tumors	Writers	Readers	Erasers	Functions
Glioma	METTL3, METTL14, WTAP	YTHDF2, IGF2BP2/3	ALKBH5	YTHDF2 and ALKBH5 promote tumor progression through metabolic disorders and oncogene activation [71, 77].
HCC	METTL3, METTL14, METTL16, WTAP	YTHDF1, IGF2BP1/2/3	FTO, ALKBH5	METTL14 exerts anticancer effects, while others promote translation and autophagy, enhancing tumor stemness [75].
PC	METTL3, METTL14, WTAP	IGF2BP1/2	FTO, ALKBH5	METTL14 promotes tumor growth and metastasis [76].
AML	METTL3, METTL14, WTAP	YTHDF2, YTHDC1, IGF2BP1/2	FTO, ALKBH5	METTL3/14, YTHDF2/YTHDC1 proteins work to maintain the activity of oncogenes, inhibit tumor suppressor genes, and drive cancer development by regulating m^6^A RNA modification [67, 68, 88].
Melanoma	METTL3, METTL14	YTHDF1, YTHDF2, IGF2BP1	FTO	Targeting METTL3/METTL14 for degradation reduces m^6^A levels and inhibits cancer cell proliferation [156].
BC	METTL3, METTL14, WTAP	YTHDF1, YTHDF2, YTHDF3, IGF2BP1/3	ALKBH5, FTO	YTHDF3 translates m^6^A-rich transcripts and promotes brain metastasis of tumors [82]; FTO regulates the stability and expression of PPARγ and C/EBP-α/β mRNA, and promotes tumor growth and lung metastasis [150].
GC	METTL3, METTL16, WTAP	YTHDF1, IGF2BP2/3	FTO	METTL3-mediated HDGF mRNA stabilization promotes liver metastasis [83].
CRC	METTL3, METTL14	YTHDF1, YTHDF2, IGF2BP1/2/3	FTO	METTL14 inhibits tumor proliferation by promoting the degradation of SOX4 mRNA and has an anticancer effect [85].
BLC	METTL3, METTL14, WTAP	YTHDF1, IGF2BP1	ALKBH5, FTO	METTL14 inhibits metastasis by stabilizing NOTCH1 mRNA [84].

Abbreviations: HCC, hepatocellular carcinoma; PC, pancreatic cancer; AML, acute myeloid leukemia; BC, breast cancer; GC, gastric cancer; CRC, colorectal cancer; BLC, bladder cancer.

**Table 2.** The role of RNA m^6^A modification-related regulatory factors in other diseases.

Diseases		Writers	Readers	Erasers	Functions
Metabolic diseases	Obesity	METTL3, METTL14	YTHDF1, YTHDF2, YTHDC1	FTO	The specific knockout of METTL3 decreases m^6^A levels and inhibits critical thermogenic genes, which worsens obesity induced by a high-fat diet [93].
	NAFLD	METTL3, METTL14	YTHDF2, YTHDC2	ALKBH5, FTO	Regulating adipocyte proliferation and lipid synthesis [98, 101, 103].
	Diabetes	METTL3, METTL1, WTAP	-	FTO	Exacerbating hepatic steatosis and insulin resistance decreased insulin secretion and induced diabetes [104–107].
	DN	METTL3, METTL1, WTAP	YTHDF1, IGF2BP1/2	FTO	Renal interstitial fibrosis and glomerular endothelial-mesenchymal transition [108, 109].
Cardiovascular system diseases	Atherosclerosis	METTL3, METTL14	YTHDF1, YTHDF2	ALKBH5, FTO	Promote the formation and progress of atherosclerotic plaque [111, 112].
	HF	METTL3	-	FTO	Cardiac contractile dysfunction, myocardial hypertrophy, and progression of HF [117, 120].
Nervous system diseases	Ischemic stroke	-	YTHDF1	FTO	YTHDF1 promotes p65 translation to exacerbate ischemia-reperfusion inflammation; miRNA-421–3p targets YTHDF1 for neuroprotection [130].
	TBI	METTL14	-	FTO	FTO maintains the stability of post-traumatic brain function [131].
	PD	-	-	FTO	FTO regulates midbrain dopamine signaling and maintains D2-like receptor function, being crucial for dopaminergic neuron survival [134].
	AD	METTL3	-	FTO	m^6^A methylation level was significantly increased, accompanied by up-regulation of METTL3 and down-regulation of FTO [132].

Abbreviations: NAFLD, non-alcoholic fatty liver disease; DN, diabetic nephropathy; HF, heart failure; TBI, traumatic brain injury; PD, Parkinson’s disease; AD, Alzheimer’s disease.

#### The role of m^6^A in tumors

By regulating RNA stability, translation efficiency, nucleoplasmic transport, and intermolecular interactions, m^6^A modification exerts a significant influence on tumor biological capabilities such as proliferation, metastasis, and drug resistance.

##### Regulating proliferative signaling

m^6^A modification plays a vital role in regulating cell proliferation and apoptosis in tumors. For example, methyltransferases METTL3 and METTL14 enhance translation efficiency by adding m^6^A marks to MYC, a process that supports rapid proliferation in acute myeloid leukemia (AML) [67, 68]. Conversely, the demethylase FTO promotes cell survival by removing m^6^A modifications from MYC, thus enhancing its stability [69]. This mechanism is equally prevalent in solid tumors. For instance, METTL3 promotes bladder cancer (BLC) cell proliferation by methylating target mRNAs, including *AFF4, MYC*, and *pri-miR-221/222* [70]. ALKBH5 accelerates tumor growth in glioblastoma (GBM) by enhancing FOXM1 expression through the removal of m^6^A [71]. In hepatocellular carcinoma (HCC), METTL3 suppresses SOCS2 expression in a YTHDF2-dependent manner [72], while WTAP reduces *ETS1* mRNA levels through the m^6^A mechanism, collectively promoting cell cycle progression and tumor proliferation [73]. Furthermore, down-regulation of YTHDF2 reduces degradation of tumor-promoting factors *IL11* and *SERPINE2* mRNA, thereby facilitating tumor development [74]. Notably, the same regulatory factor may exert opposing effects across different cancers. For example, METTL14 inhibits HCC proliferation by promoting miR-126 maturation and inducing degradation of mRNAs like *EGFR* [75]. However, in pancreatic cancer (PC), METTL14 promotes cell proliferation by destabilizing the mRNA of p53 apoptosis effector related to PMP-22 (*PERP*) [76]. Additionally, m^6^A modifications confer sustained survival and growth advantages by altering tumor cell metabolic states. In highly malignant glioblastoma, YTHDF2 disrupts cholesterol metabolism by recognizing m^6^A modifications on the key cholesterol metabolism factor LXRα and facilitating its degradation, significantly promoting tumor cell proliferation [77].

##### Modulating invasion and metastasis

Tumor metastasis is associated with the combined effects of multiple mechanisms, directly impacting patient prognosis. As tumors progress to higher stages, m^6^A modification drives disease spread by enhancing the metastasis and invasion of cancer cells. Low METTL3 expression in renal cell carcinoma (RCC) suggests a tumor suppressor role [78], while reduced METTL14 expression weakens m^6^A modification on tumor suppressor transcripts (e.g. BPTF and PTEN), thereby promoting cancer cell invasion and metastasis [79]. In colorectal cancer (CRC), METTL3 promotes malignant transformation and metastasis by facilitating the methylation and maturation of pri-miR-1246 [80]. Moreover, due to hypermethylation of specific sites in CRC, YTHDF2/3 degrades the lncRNA CARMN, thereby relieving inhibition of the downstream oncogenic axis and accelerating tumor progression [81]. Alternatively, YTHDF3 can promote brain metastasis and growth of breast cancer (BC) cells by preferentially translating m^6^A-rich transcripts such as ST6GALNAC5 and EGFR [82]. Notably, highly expressed METTL3 promotes gastric cancer (GC) cell liver metastasis by stabilizing *HDGF* mRNA via m^6^A [83]. In contrast, METTL14 inhibits metastasis by stabilizing *NOTCH1* mRNA in BLC and promoting the degradation of mRNAs, including *SOX4* in CRC [84, 85]. These studies highlight the context-dependent roles of m^6^A.

##### Adjusting drug resistance and stem cell properties

Molecular adaptation mediated by m^6^A is a key factor in tumor treatment resistance and the maintenance of stem cell characteristics. For example, METTL3 upregulates lactate dehydrogenase A (LDHA) through a dual mechanism, thereby enhancing glycolytic activity in CRC and conferring 5-fluorouracil (5-FU) resistance. It promotes LDHA transcription by stabilizing hypoxia-inducible factor (*HIF-1α*) mRNA and enhances its translation by recruiting YTHDF1 protein through methylation of the LDHA CDS region [86]. In glioma, METTL3 and IGF2BP3 stabilize the mRNA of cytoplasmic polyadenylation element binding protein 2 (*CPEB2*) through m^6^A modification, which activates the SRSF5/ETS1 pathway to upregulate tight junction proteins (ZO-1, occludin, tight junction protein-5), reducing blood-tumor barrier permeability and causing chemoresistance [87]. m^6^A also plays a crucial role in maintaining tumor stem cell properties by regulating RNA metabolism and function. For instance, YTHDF2 preserves leukemia stem cell characteristics by extending the lifespan of stem cell fate-related mRNAs [88]. Similarly, inhibiting FTO weakens cancer cell self-renewal capacity by blocking m^6^A modification on mRNAs associated with ovarian cancer (OC) stem cell properties [89]. These findings collectively underscore the central role of m^6^A modification in tumor therapeutic resistance and the maintenance of stem cell properties.

Remarkably, the m^6^A regulatory network contains key nodes with dual or multiple functions. For example, YTHDC1 enhances the malignant regulatory networks in leukemia by protecting m^6^A-modified mRNA from degradation [90], while also inhibiting aerobic glycolysis and tumorigenesis in PC by promoting miR-30d maturation [91]. This context-dependent functional difference highlights the complexity of the m^6^A regulatory network, presenting both challenges and opportunities for precision targeted therapy.

#### The role of m^6^A in metabolic diseases

m^6^A modification is critical for regulating systemic metabolism. Its dysregulation disrupts the balance of cellular and systemic metabolism by interfering with key regulators of lipid, glucose, and energy homeostasis. This disruption is closely connected to obesity, nonalcoholic fatty liver disease (NAFLD), and type 2 diabetes mellitus (T2D), ultimately promoting the occurrence and development of these major metabolic diseases.

##### Affecting lipogenesis and liver lipid metabolism

m^6^A modification plays a crucial role as a regulator of lipogenesis and energy metabolism. For instance, knockdown of METTL3 in bone marrow mesenchymal stem cells promotes adipocyte differentiation by stabilizing Janus kinase 1 (*JAK1*) mRNA, which activates the signal transducer and activator of transcription 5 (STAT5) pathway and enhances the expression of the adipogenic transcription factor C/EBP-β [92]. On the other hand, in brown adipose tissue, which is essential for energy metabolism, specific knockout of *METTL3* reduces m^6^A levels and suppresses key thermogenic genes, thereby exacerbating obesity and insulin resistance induced by a high-fat diet [93]. Alternatively, the reader protein YTHDC1 supports the normal development and function of brown fat by directly binding to PPARγ and preventing its ubiquitin-mediated degradation [94]. FTO similarly regulates adipocyte-related pathways in an environment-dependent manner. In white adipose tissue, the specific knockout of *FTO* increases the m^6^A modification of *HIF1A* mRNA, improving its translation efficiency, and activating heat-related genes [95]. This process encourages the transformation of white fat into a beneficial brown fat-like phenotype, thereby increasing energy expenditure to combat obesity [96]. In contrast, inhibiting FTO blocks adipocyte differentiation by enhancing the m^6^A modification of the adipogenic factor RUNX1 partner transcriptional co-repressor 1(*RUNX1T1*) mRNA or via YTHDF2-mediated degradation of *ATG5* and *ATG7* mRNA [43, 97].

##### Impacting liver lipid metabolism

The m^6^A regulatory network is important for maintaining liver lipid metabolism balance, and its disruption directly contributes to the progression of non-alcoholic fatty liver disease (NAFLD). In hepatocytes, METTL3 deficiency has a protective effect against metabolic syndrome by extending the half-life of *LPIN1* mRNA, a vital factor in lipid metabolism [98]. Meanwhile, overexpression of METTL3 hinders autophagy by stabilizing *Rubicon* mRNA, impeding the clearance of lipid droplets, and exacerbating liver fat accumulation [99]. Furthermore, METTL3 synergizes with METTL14 to upregulate the protein levels of lipid synthases, such as ACLY and stearoyl-CoA desaturase 1 (SCD1), thereby directly stimulating the synthesis of triglycerides and cholesterol. This process subsequently triggers chronic inflammation, apoptosis, and DNA damage in the liver, ultimately accelerating the progression of NAFLD and HCC [100]. Demethylases also play a significant role: FTO enhances the stability and lipid synthesis activity of transcription factors sterol regulatory element binding transcription factor 1 (SREBF1) and carbohydrate responsive element binding protein (ChREBP1) by reducing their m^6^A levels, and thus promotes liver steatosis [101]. Similarly, ALKBH5 increases the stability of lncRNA LINC01468, promoting the degradation of Src homology 2 domain-containing inositol 5’-phosphatase (SHIP2) protein and ultimately activating the PI3K/AKT/mTOR pathway to enhance liver lipid synthesis [102]. In addition, the reader protein YTHDC2 reduces the mRNA stability of multiple lipid synthesis genes, thereby alleviating liver steatosis [103].

##### Influencing pancreatic β-cell function and glucose homeostasis

Dysregulation of m^6^A modification can impair pancreatic β-cell function and glucose homeostasis, contributing to the development of related diseases like T2D. Loss of function of METTL3, METTL14, or WTAP results in the downregulation of insulin secretion-related genes and β-cell-specific transcription factors, causing β-cell dysfunction and inadequate insulin secretion [104–106]. The demethylase FTO influences insulin secretion and hepatic gluconeogenesis by enhancing mRNA translation of key metabolic factors such as FOXO1 and G6PC [107]. In high glucose environments, m^6^A modification mediated by WTAP and METTL3 stabilizes target mRNAs like NLR family pyrin domain containing 3 (*NLRP3*) and TIMP metallopeptidase inhibitor 2 (*TIMP2*) by recruiting the reader proteins IGF2BP1 and IGF2BP2. This process activates the NLRP3 inflammasome and the Notch signaling pathway, ultimately leading to podocyte injury and diabetic nephropathy [108, 109].

#### The role of m^6^A in cardiovascular diseases

m^6^A modification participates in regulating cardiovascular disease processes. In atherosclerosis, m^6^A drives disease progression by influencing key cellular events such as endothelial inflammation, macrophage polarization, and plaque stability [110]. In addition, m^6^A is also implicated in the pathological regulation of hypertension, heart failure, ischemic heart disease, and pulmonary hypertension.

##### Atherosclerosis

Many studies have demonstrated that m^6^A modification is a key regulatory mechanism in the progression of atherosclerosis. For instance, METTL14 directly binds to *FOXO1* mRNA, increasing its m^6^A levels and enhancing translation efficiency via YTHDF1 [111]. This process drives endothelial inflammation and plaque formation. Likewise, METTL3 also promotes m^6^A modification and translation of *BRAF* mRNA in a YTHDF1-dependent manner, worsening hyperlipidemia-induced vascular inflammation [112]. Additionally, METTL3 enhances monocyte adhesion to the endothelium by mediating m^6^A-dependent degradation of *PGC-1α* mRNA [113]. It also upregulates the pro-inflammatory factor NLRP1 while inhibiting the protective factor KLF4 by modifying their respective mRNAs, leading to TNF-α-mediated endothelial dysfunction [113]. Conversely, METTL3 knockdown alleviates oxidized low-density lipoprotein (ox-LDL)-induced endothelial damage by downregulating NPC1L1. This intervention also significantly reduces arterial plaques associated with a high-fat diet [114]. On another front, overexpression of FTO significantly reduces plasma total cholesterol and the accumulation of cholesterol esters in macrophages containing oxidized LDL, thereby preventing the formation of atherosclerotic plaques [115].

##### Hypertension and heart diseases

In the context of hypertension and myocardial remodeling, FTO inhibits lipocalin-type prostaglandin D synthase (L-PGDS) in blood vessels through an m^6^A-dependent mechanism. This inhibition reduces prostaglandin D2(PGD2) production, leading to increased vascular resistance and promoting disease progression [116]. Overexpression of METTL3 promotes compensatory cardiac hypertrophy without impacting cardiac function [117]. In contrast, METTL3 knockout accelerates pathological cardiac hypertrophy and heart failure by reducing m^6^A levels of poly (ADP-ribose) polymerase family member 10 (*Parp10*) mRNA and increasing PARP10 expression [117, 118].

m^6^A levels are significantly enhanced in myocardial tissue with heart failure (HF), primarily affecting pathways related to glycolysis, mitochondrial function, and fibrosis [119, 120]. In stress-induced HF models, downregulation of FTO expression leads to increased m^6^A modification of phosphoglycerate mutase 2 (*Pgam2*) mRNA, which impairs myocardial glycolysis and systolic function [121]. Conversely, FTO overexpression enhances cardiac function and protects against cardiac remodeling by regulating m^6^A on contractile-related transcripts such as sarcoplasmic/endoplasmic reticulum calcium ATPase 2a (Serca2a) and ryanodine receptor 2 (Ryr2) [122].

For ischemic heart disease, METTL3 decreases the stability of transcription factor EB (*TFEB*) mRNA by increasing its m^6^A modification. This inhibitory effect disrupts the autophagic flux in hypoxic cardiomyocytes and promotes apoptosis [123]. In valvular heart disease, METTL3 reduces TWIST1 expression in an m^6^A-dependent manner, promoting osteogenic differentiation of human aortic valve interstitial cells and accelerating valve calcification [124]. Notably, a rat model of pulmonary hypertension exhibits significantly elevated total mRNA m^6^A levels, suggesting m^6^A modification may provide key insights into this condition [125]. Studies demonstrate that circRNAs such as circXpo6 and circTmtc3 participate in the pathogenesis of pulmonary hypertension through the m^6^A regulatory network [126].

#### The role of m^6^A in nervous system diseases

m^6^A modification plays an important regulatory role in the central nervous system (CNS), with the brain exhibiting the highest levels of RNA m^6^A methylation *in vivo* [127, 128]. This mechanism drives key physiological processes, including brain volume regulation, memory consolidation, and postnatal cortical neurogenesis in mammals [129]. Abnormal m^6^A levels are directly linked to the development of various neurological diseases.

##### Brain injuries

Different types of acute brain injuries exhibit distinct m^6^A regulatory patterns. In the transient focal cerebral ischemia model, YTHDF1 enhances p65 mRNA translation via m^6^A modification, aggravating the inflammatory response following cerebral ischemia-reperfusion [130]. Whereas miRNA-421–3p counteracts this effect and exerts neuroprotection by directly targeting YTHDF1 [130]. After traumatic brain injury (TBI), the expression of METTL14 and FTO is significantly reduced, and further inhibition of FTO exacerbates neurological deficits, indicating the crucial role FTO plays in maintaining post-traumatic brain functional stability [131].

##### Neurodegenerative diseases

The development of neurodegenerative diseases is closely associated with m^6^A modification. In the mouse model of Alzheimer’s disease (AD), m^6^A methylation levels in the cerebral cortex and hippocampus RNA are significantly increased, accompanied by upregulation of METTL3 and downregulation of FTO [132]. The survival of dopaminergic neurons in Parkinson’s disease (PD) is critically dependent on m^6^A levels, which are regulated by FTO through its role in regulating midbrain dopamine signaling and maintaining the normal function of dopamine D2-like receptors [133, 134].

### m^6^A-related targeted therapy

Given the critical role of m^6^A modification in diseases such as cancer, metabolic disorders, and neurological conditions, targeting this mechanism has become a highly promising therapeutic strategy. This approach primarily utilizes small-molecule drugs or modulators that act on m^6^A regulatory factors to modulate RNA splicing, stability, and translation, thereby achieving therapeutic outcomes. Consequently, m^6^A and its associated regulatory factors are considered potential biomarkers for diagnosis and prognosis, while also serving as important therapeutic targets (Table 3).

**Table 3.** Targeted drugs for m^6^A-modified regulators.

Diseases	Roles	Regulators	Target drugs
Tumors	Writer	METTL3	STM2457, UZH2, UZH1a [153, 155].
	Eraser	FTO	Rhein, MA/MA2, R-2HG [143, 146, 148, 149].
	Reader	IGFBP1	BTYNB [157].
		IGFBP2	JX5 [158].
		YTHDF2	Curcumin [159].
Nervous system diseases	Eraser	FTO	Entacapone [144].
Diabetic	Writer	METTL3	Exenatide, the total flavone of okra [141, 142].
	Writer	METTL14	Gan Jiang Ling Zhu Decoction [152].
	Eraser	FTO	Dac51 [147].
		ALKBH5	Maslinic acid [160].

#### As biomarkers for diseases

Alterations in the levels of m^6^A modification or abnormal expression of its associated enzymes have demonstrated potential as biomarkers for various diseases. Previous studies revealed that the m^6^A levels in peripheral blood leukocytes of non-small cell lung cancer (NSCLC) patients were significantly elevated, with the degree of increase correlating with tumor stage and differentiation. This suggests that m^6^A levels may serve as a potential non-invasive diagnostic indicator and a means for monitoring treatment efficacy [135]. Similarly, CRC patients exhibited significantly higher m^6^A levels in peripheral blood immune cells compared to healthy individuals, and combining this metric with traditional tumor markers further enhanced diagnostic accuracy. The m^6^A index was also linked to tumor metastasis and showed a decrease following treatment [136]. Notably, the m^6^A reader protein YTHDF1 is generally overexpressed in various cancer tissues, including lung cancer and GC. Due to its high sensitivity and specificity, YTHDF1 is poised to become a valuable diagnostic marker for pan-cancer applications, with its expression level closely associated with patient prognosis [137]. Furthermore, the abnormal expression of m^6^A demethylases FTO and ALKBH5 has been implicated in the onset and progression of lung cancer, positioning them as potential targets for diagnosis and prognosis evaluation [138].

The abnormal expression or altered activity of m^6^A regulatory factors serves as an important prognostic indicator in various cancers. For example, METTL3, highly expressed in GC and CRC, serves as an independent predictor of poor prognosis [83, 139]. Its expression level is significantly higher in advanced GC compared to early-stage cancer, and combining METTL3 expression with tumor, node, and metastasis (TNM) staging improves the accuracy of prognostic assessment [140].

#### As therapeutic targets for diseases

Targeting m^6^A pathways shows considerable therapeutic potential across a range of diseases. In the context of metabolic diseases, several drugs have been shown to exert therapeutic effects through the modulation of m^6^A modification. For instance, okra flavonoids significantly attenuate high glucose-induced podocyte pyroptosis by inhibiting METTL3-mediated m^6^A modification of *PTEN* mRNA, thereby suppressing NLRP3 inflammasome activation [141]. The GLP-1 receptor agonist Exenatide effectively improves H_2_O_2_-induced apoptosis in mouse pancreatic β-cells by restoring m^6^A methylation levels via METTL3 targeting [142]. Additionally, the FTO inhibitor Dac51 can rescue NR3C1-mediated β-cell dysfunction and hyperglycemia by suppressing abnormal autophagy, highlighting its therapeutic potential for diabetes and related complications [143].

In PD treatment, the drug entacapone directly binds to and inhibits FTO activity, significantly enhancing liver glucose metabolism and adipose tissue thermogenesis via activation of the FTO-FOXO1 axis [144]. Furthermore, melatonin, a circadian rhythm regulator, elevates m^6^A modification in adipocytes, promotes resistin mRNA degradation, and reduces extracellular vesicles formation, thereby alleviating hepatic endoplasmic reticulum (ER) stress-induced steatosis [145].

FTO inhibitors also play a central role in tumor therapy. For example, Rhein increases the sensitivity of leukemia cells to tyrosine kinase inhibitors [146], while MA2 can inhibit the self-renewal of GSCs [147, 148]. Notably, R-2-hydroxyglutarate (R-2HG) competitively binds to the Fe^2+^ site in the active center of FTO, thereby regulating m^6^A modification of mRNAs such as *MYC* and *PFKP*, and inhibiting the proliferation and aerobic glycolysis of AML and GBM cells [69, 149]. FTO inhibitors have shown significant efficacy in inhibiting tumor growth. Specifically, an inhibitor named 18 097 reduces mRNA stability and expression of PPARγ and C/EBP-α/β by inhibiting FTO, leading to decreased lipid uptake and oxidation in cancer cells, and effectively restraining breast cancer growth and lung metastasis *in vivo* [150].

Natural products and traditional Chinese medicines also contribute to m^6^A-related therapies. For instance, betaine enhances autophagy and inhibits liver cancer stem cell traits by increasing m^6^A modification on autophagy-related gene 3 (*ATG3*) mRNA and facilitating YTHDF1 binding, which in turn stabilizes ATG3 transcripts [151]. Furthermore, Ganjiang Lingzhu Decoction alleviates liver inflammation and steatosis in mice by promoting m^6^A modification of *Ugt2a3* mRNA in a METTL14-dependent manner [152]. Studies have also confirmed that SAM analogs and STM2457 effectively target METTL3 in AML, leading to significantly enhanced apoptosis of leukemia cells [153]. Similarly, saikosaponin D induces apoptosis in AML by blocking FTO-mediated up-regulation of tRNA m^6^A modification [154]. Besides, inhibitors such as RM3 and UZH2 suppress tumor progression in melanoma, AML, and prostate cancer (PCa) by targeting the METTL3/METTL14 complex and reducing m^6^A modification levels [155, 156]. BTYNB and JX5, as IGF2BP2 inhibitors, can also exert inhibitory effects on tumor cell proliferation. In addition to small molecule compounds, some plant extracts also play an important role in inhibiting tumors [157, 158]. Curcumin inhibits YTHDF2 expression, downregulates mRNA levels modified by m^6^A, and suppresses prostate cancer cell proliferation and migration [159]. In inflammatory injury, plant extract Maslinic acid promotes ALKBH5 recruitment of TXNIP mRNA in high glucose-induced HUVECs, enhances its m^6^A demethylation, reduces TXNIP mRNA stability and expression, inhibits reactive oxygen species (ROS) and pro-inflammatory factors such as TNF-α, IL-6, IL-1β, and alleviates endothelial inflammation and injury [160].

## Other methylation modifications

Beyond the well-characterized m^6^A, other RNA methylation modifications, including m^6^Am, m^7^G, m^5^C, and m^1^A constitute another layer of post-transcriptional regulation. They are similarly reversible and governed by dedicated writers, erasers, and readers, thereby forming intricate regulatory networks that collectively influence mRNA fate and function (Table 4).

**Table 4.** Functions of RNA methylation modification regulatory factors.

Methylation	Roles	Regulators	Functions
m^6^A	Writer	METTL3	Combine SAM and catalyze the transmission of adenosine [34].
		METTL14	Forms a heterodimer with METTL3 [35].
		METTL5/TRMT112	Methylate 18S rRNA [41].
		METTL16	Methylate MAT2A mRNA and U6 snRNA [39].
		WTAP	Regulatory factors [37].
		ZC3H13	Regulatory factors [32].
		ZCCHC4	Catalyze m^6^A in some specific RNAs [40].
		KIAA1429	Regulatory factors [33].
	Eraser	FTO	Removing m^6^A modifications from specific transcripts [45].
		ALKBH5	Targeting m^6^A modification on RNA [44].
	Reader	YTHDF1	Promote translation efficiency, enhance ribosome loading [48].
		YTHDF2	Takes the target mRNA to degradation [49].
		YTHDF3	Works synergistically with YTHDF1 [51].
		YTHDC1	Binds to m^6^A and increases shear factor [53].
		YTHDC2	Recognize m^6^A modification [52].
		IGF2BPs	Collect RNA stabilizers [55].
		hnRNPs	Interact with YTH and IGF2BP [57, 58].
m^6^Am	Writer	PCIF1	Formation m^6^Am next to the 5’ cap of mRNAs and in snRNAs [252].
		METTL4	Formation of m^6^Am within position 30 in the U2 snRNA [254, 257].
	Eraser	FTO	Demethylates m^6^Am to Am [258].
	Reader	PCF11	Identify and combine m^6^Am on mRNA [259].
m^1^A	Writer	TRMT6/TRMT61A	Promote the formation of m^1^A58 on cytoplasmic tRNA [201].
		TWMT61B	Regulate the methylation of mitochondrial tRNA [200].
		TRMT10C	Regulate the methylation of mitochondrial mRNA [195].
	Eraser	ALKBH1	Facilitate the demethylation of m^1^A in tRNA [202].
		ALKBH3	Demethylate m^1^A and m^3^C in tRNA [205].
		FTO	Inhibit translation by catalyzing the demethylation of m^1^A tRNA [199].
	Reader	YTHDF2	Recognizes both m^6^A and m^1^A [207].
		YTHDF3	Inhibits cell invasion by degrading IGF1R mRNA [209].
		YTHDC1	Prevent methyltransferase-induced DNA fragmentation [210].
m^5^C	Writer	NSUN1	Catalyze m^5^C modification of rRNA [163, 164].
		NSUN2	Catalyze m^5^C modification of various RNAs [165, 166].
		NSUN3	Catalyze m^5^C modification of mitochondrial tRNA [167].
		NSUN4	Catalyze m^5^C modification of rRNA in mitochondria [168].
		NSUN5	Act on rRNA and specific mRNA sites in the nucleus [169, 170].
		NSUN6	Act on rRNA and specific mRNA sites in the nucleus [169, 170].
		DNMT2	Catalyzes the m^5^C modification of tRNA [165, 171].
	Eraser	TET1/2/3	Demethylation by oxidizing 5-methylcytosine [173, 183].
		ALKBH1	Influences the biosynthesis of 5hmC and f5C in both cytoplasmic and mitochondrial tRNA [179].
	Reader	ALYREF	Promotes the nuclear export of m^5^C-modified rRNAs [181].
		YBX1	Recognize m^5^C [182].
		FMRP	Mediated demethylation of m^5^C RNA modification [183].
m^7^G	Writer	METTL1/WDR4	Stabilizing the three-dimensional structure of tRNA via m^7^G46 [223, 224].
		RNMT/RAM	Catalyze the m^7^G into the mRNA 5’-cap [228, 229].
		TRMT112/WBSCR22	Participates in the m^7^G modification of 18s rRNA [231, 232].
	Reader	eIF4E	Identify m^7^G cap [234].
		CBC	Identify m^7^G cap [235].
		QKI	Specifically recognizes m^7^G-modified internal mRNA [237, 238].

### m^5^C

**Figure 3.**
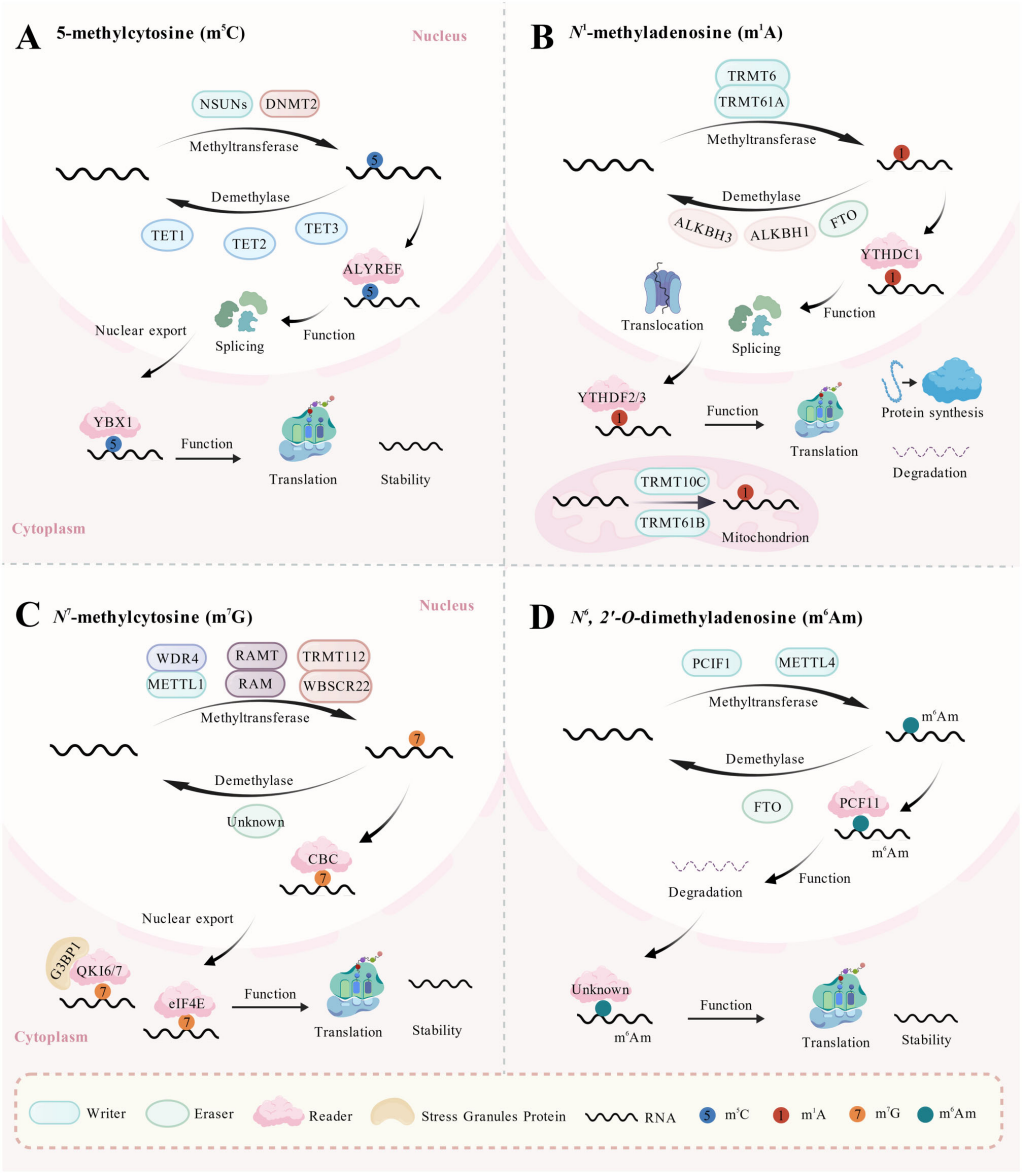
The dynamic regulation and functional roles of other types of RNA methylation. (**A**) 5-methylcytosine (m^5^C): m^5^C modification is primarily catalyzed by writers, including members of the NSUN family and DNMT2. The demethylation process is mediated by TET1/2/3 enzymes, which act as erasers. Readers such as ALYREF and YBX1 specifically recognize m^5^C sites, mediating critical biological processes and broadly influencing post-transcriptional gene regulation. (**B**) *N*^1^- methyladenosine (m^1^A): The m^1^A modification is dynamically regulated by writer and eraser enzymes. In the nucleus, the TRMT6/TRMT61A complex catalyzes m^1^A deposition on tRNA, whereas in mitochondria, TRMT61B and TRMT10C catalyze m^1^A modification on tRNA and mRNA, respectively. The demethylation process is facilitated by eraser enzymes, including FTO, ALKBH1, and ALKBH3. m^1^A-modified RNA is recognized by reader proteins such as YTHDF1-3 and YTHDC1. (**C**) *N*^7^-methylcytosine (m^7^G): The m^7^G modification is dynamically regulated by a suite of writer complexes: METTL1/WDR4 complex catalyzes m^7^G methylation on tRNA and mRNA, WBSCR22/TRMT112 modifies 18S rRNA, and RNMT/RAM acts on mRNA caps. Modified RNA is recognized by reader proteins, including eIF4E, CBC, and QKI. (**D**) *N*^6^, *2’*-*O*-dimethyladenosine (m^6^Am): PCIF1 has been identified as the writer for mRNA m^6^Am modification, while METTL4 primarily methylates U2 snRNA. The eraser protein FTO reverses m^6^Am, making it a reversible mark. Reader proteins such as PCF11 recognize and bind to the m^6^Am-modified RNA.

m^5^C is a key element of RNA post-transcriptional modification, directly influencing mRNA stability, nucleoplasmic transport, and translation efficiency by regulating the methylation of the fifth carbon atom of cytosine. This modification ultimately enhances the proliferation, migration, and invasion of tumor cells (Fig. 3A).

#### Dynamic regulation of m^5^C modification

The known m^5^C methyltransferases primarily include members of the NSUN family (NSUN1-7) and DNA methyltransferase 2 (DNMT2), each exhibiting distinct substrate specificity and cellular localization [161, 162]. For example, NSUN1 predominantly catalyzes m^5^C modification of rRNA within the nucleus [163, 164]. NSUN2, the most extensively studied member of the NSUN family, is primarily nuclear-localized and targets multiple m^5^C sites in tRNA [165]. Additionally, it participates in the methylation of various RNAs [166]. NSUN3 and NSUN4 are responsible for modifying tRNA and rRNA in mitochondria, respectively [167, 168], while NSUN5 and NSUN6 primarily target rRNA and specific mRNA sites in the nucleus [169, 170], with NSUN6 specifically recognizing the TCCA motif within stem-loop structures [170]. Notably, although DNMT2 is classified as a DNA methyltransferase, it also catalyzes m^5^C modification of tRNA [165, 171].

The erasers of m^5^C have also been identified, including the ten-eleven translocation (TET) family and alkB homolog 1 (ALKBH1). The TET family, consisting of TET1, TET2, and TET3, is involved not only in DNA demethylation but also in RNA demethylation [172, 173]. Their catalytic domain oxidizes 5-methylcytosine (5mC) to 5-hydroxymethylcytosine (5hmC), 5-formylcytosine (5fC), and 5-carboxycytosine (5caC) [174, 175]. Studies indicate that TET-mediated deposition of 5hmC decreases mRNA stability [176, 177]. Structurally, the N-termini of TET1 and TET3 contain a CXXC zinc finger domain, which may work as a functional unit directing these enzymes to specific genomic regions for action, whereas TET2 lacks this feature [178]. Research on ALKBH1 demethylation is currently focused primarily on m^6^A and m^1^A rather than m^5^C. However, studies have shown that ALKBH1 can influence the biosynthesis of 5hmC and f5C at the first anticodon position (position 34) of cytoplasmic and mitochondrial tRNA [179]. Further investigation is required to clarify the roles of the TET family and ALKBH1 in these oxidative conversions and to determine whether the functional interplay exists between them.

#### Modulatory effects of m^5^C modification

Currently known m^5^C reader proteins include ALY/REF export factor (ALYREF), Y-box binding protein 1 (YBX1), and fragile X mental retardation protein (FMRP) [180], which influenc downstream RNA functions by interacting with RNA modified with m^5^C. Among these, ALYREF facilitates the nuclear export of m^5^C-modified mRNAs [181]. YBX1 specifically recognizes m^5^C through tryptophan residues within its cold shock domain and collaborates with the ELAVL1 protein to maintain mRNA stability [182]. As another m^5^C reader, FMRP cooperates with TET1 to mediate the demethylation of m^5^C in DNA-RNA hybrids, thereby promoting transcriptional homologous recombination [183].

#### The role of m^5^C in diseases

The m^5^C modification plays a dual regulatory role in viral infection and the stress response. Under normal stress conditions, NSUN2 deficiency leads to intracellular accumulation of unmethylated non-coding RNAs such as RPPH1 and 7SL [184], which activates the retinoic acid-inducible gene I (RIG-I) mediated interferon signaling pathway and suppresses viral proliferation [184]. However, during hepatitis B virus (HBV) infection, this regulatory mechanism is disrupted: m^5^C modification at specific sites of *HBV* mRNA enhances the translation of the hepatitis B virus X protein (HBx), while HBV infection downregulates NSUN2 expression, resulting in reduced m^5^C levels of interferon (*IFN*) mRNA [185]. These changes collectively suppress interferon production and promote viral replication [185]. At the immune regulation level, NSUN2-mediated m^5^C modification inhibits the cGAS-STING pathway, and thus interfering with interferon production, promoting colorectal cancer progression, and contributing to resistance to PD-L1 immunotherapy [186]. In addition, the FMRP-TET1 axis regulates m^5^C modification during DNA damage repair, leading to tumor cell radioresistance via a BRCA-independent manner [183].

During tumorigenesis, m^5^C modification directly drives abnormal proliferation of tumor cells by regulating the expression of key oncogenic genes. For instance, high levels of NSUN2 significantly enhance CRC proliferation by stabilizing the mRNA of the proto-oncogene *SKIL1* [187]. Similarly, NSUN2 overexpression promotes the development of esophageal squamous cell carcinoma (ESCC) by stabilizing mRNAs involved in the PI3K/Akt and MAPK signaling pathways [188]. This proliferative mechanism is also observed in retinoblastoma, where NSUN2 accelerates tumor growth by up-regulating the purine synthesis-related protein PFAS [189]. In CRC, m^5^C modification drives tumor metabolic reprogramming through the NSUN2/YBX1/m^5^C-ENO1 signaling axis, establishing a self-sustaining positive feedback loop [190]. Specifically, NSUN2-mediated m^5^C modification enhances the stability of *ENO1* mRNA, while YBX1, as the reader protein, recognizes this modification and promotes glycolytic pathway reprogramming and lactate production. Accumulated lactate further induces histone H3K18 lactylation and NSUN2 lactylation at the K356 site, which in turn activates NSUN2 transcription and enhances its RNA-binding capacity. This mechanism forms a continuously activated m^5^C-mediated metabolic-epigenetic cycle that promotes CRC progression [190], highlighting the essential role of m^5^C modification in sustaining energy metabolism within tumors. Beyond promoting cell proliferation, m^5^C also critically regulates tumor invasion and metastasis. In glioma, NSUN2-mediated m^5^C modification in the 3’-UTR of *ATX* mRNA strengthens its interaction with the reader protein ALYREF [191], facilitating nuclear export of the transcript and promoting glioma cell migration. ALYREF also stabilizes *NOTCH1* mRNA in an m^5^C-dependent manner, activating the Notch signaling pathway and directly driving the metastasis of nasopharyngeal carcinoma (NPC) [192].

Collectively, these findings suggest that m^5^C modification, as a multifunctional RNA epigenetic regulatory mechanism, is vital in various biological processes, including viral infection, tumorigenesis, and immune regulation, making it a promising therapeutic target.

### m^1^A

m^1^A, which refers to the methylation of the first nitrogen atom of adenosine (Fig. 3B), is predominantly enriched in the 5’-UTR of mRNA, particularly around the translation initiation site. In addition to mRNA, m^1^A modifications are widely present in tRNA, rRNA, and lncRNA [193, 194]. This modification enhances RNA stability, promotes protein synthesis, and influences gene expression by altering the electrostatic distribution of RNA molecules and affecting base pairing [195]. Notably, m^1^A is closely related to m^6^A. Under alkaline conditions, m^1^A can be converted into m^6^A through the Dimroth rearrangement [196, 197]. Furthermore, the two modifications share common regulatory factors, including YTHDF1-3 and FTO [198, 199].

#### Dynamic regulation of m^1^A modification

The four major m^1^A methyltransferases include tRNA methyltransferase 6 (TRMT6), TRMT61A, TRMT61B, and TRMT10C. TRMT61B and TRMT10C are found in mitochondria, where they catalyze m^1^A formation at position 58 of mitochondrial tRNA and in mitochondrial *ND5* mRNA, respectively [195, 200]. In the cytoplasm, the TRMT6/TRMT61A complex facilitates the formation of m^1^A58 on tRNA, with TRMT61A serving as the catalytic subunit and TRMT6 responsible for tRNA binding [201]. The demethylation process of m^1^A is primarily mediated by ALKBH1, ALKBH3, and FTO. ALKBH1 demethylates m^1^A in tRNA, resulting in decreased translation initiation and reduced tRNA utilization in protein synthesis [202]. ALKBH3, currently the only known m^1^A demethylase for mRNA [203], recognizes and removes m^1^A and *N*^3^-methylcytosine (m^3^C) modifications [204, 205], and has been shown to regulate cancer cell glycolysis in a demethylation activity-dependent manner [206]. Structural analysis has revealed that the substrate selectivity of ALKBH3 is governed by key residues within its active site. Intriguingly, mutating Thr133 in ALKBH3 to the corresponding residue found in FTO shifted the substrate selectivity of ALKBH3 from m^1^A to m^6^A [204].

#### Modulatory effects of m^1^A modification

Studies have found that YTHDF1-3 and YTHDC1 serve multiple functions as readers of m^1^A in RNA [198]. YTHDF2 recognizes both m^6^A and m^1^A modifications and promotes rapid mRNA degradation in a synergistic manner [207], and it has also been shown to destabilize m^1^A-modified RNA [208]. In the cytoplasm, YTHDF3 inhibits trophoblast cell migration and invasion by promoting the degradation of m^1^A-methylated *IGF1R* mRNA [209]. In the nucleus, YTHDC1 collaborates with the THO complex (THOC) to prevent DNA breakage induced by m^1^A methyltransferase [210]. In contrast, the role of reader protein YTHDF1 as an m^1^A reader remains unclear. Current evidence suggests that YTHDF1 may not function independently but rather requires the formation of complexes with other proteins to exert its effects. The potential interactions among YTHDF1-3 also warrant further investigation.

#### The role of m^1^A in diseases

The m^1^A modification significantly influences various disease processes by modulating gene expression and cellular functions. First, the m^1^A modification plays a crucial role in regulating cell fate. In the tumor context, the demethylase ALKBH3 promotes cancer progression by demethylating tRNA, which enhances its cleavage into tRNA-derived small RNAs (tDRs). The tDRs in turn stabilize ribosome assembly and interact with cytochrome *c* (Cyt *c*) to suppress apoptosis [205]. The dysregulation of m^1^A modification is critical to the malignant progression of tumors. During tumorigenesis, TRMT6 enhances tRNA m^1^A modification, thereby promoting protein synthesis and cell proliferation in CRC [211]. Second, m^1^A modification acts as a key regulator in maintaining stem cell properties and functions. It supports the rapid translation requirements of HSCs by regulating T cell homeostasis and the mTORC1 signaling pathway [212, 213]. Similarly, m^1^A modification effectively sustains the self-renewal capacity of cancer stem cells by activating the Hedgehog signaling pathway and promoting PPARδ translation in liver cancer [214]. Furthermore, m^1^A serves as an important molecular mechanism enabling cells to respond to internal and external environmental stimuli. Studies indicate that tRNA-m^1^A58 levels are highly sensitive to environmental pressures. For example, Gram-positive bacteria exhibit a significant increase in this modification under heat stress [215], whereas low glucose treatment upregulates ALKBH1 expression in HeLa cells and inhibits translation via demethylation [202].

#### m^1^A-related targeted therapy

Proteins involved in m^1^A regulation, including writers, erasers, and readers, represent promising biomarkers and therapeutic targets for various diseases. Enzymes of the AlkB family, in particular, are considered attractive targets for anticancer therapy. The natural product Rhein, for instance, inhibits AlkB repair enzymes by competitively binding to the enzyme’s active site with 2-oxoglutarate (2OG), leading to increased accumulation of intracellular methylation damage such as m^1^A and m^3^C [216]. Studies have shown that TRMT61A promotes cancer in CRC by activating the m^1^A-ONECUT2-SOS1-MAPK/ERK pathway [217], suggesting that targeting TRMT61A may offer a viable therapeutic strategy for CRC. The m^5^C modification influences cell transcription and enhances tumor cell survival via the NSUN2-YBX1-QSOX1 axis, which contributes to the intrinsic resistance of EGFR mutant NSCLC to gefitinib [218]. This study highlights the potential of m^5^C as a therapeutic target for overcoming drug resistance. Interestingly, while TRMT61B depletion induces senescence in melanoma cells with low aneuploidy, it triggers apoptosis in highly aneuploid cells [219], highlighting its potential as a biomarker and therapeutic target for highly aneuploid cancers [219]. On the reader side, YTHDC1 has been identified as a direct target of miR-16–5p and plays a critical role in vascular pathology. Inhibition of miR-16–5p upregulates YTHDC1 expression, which enhances smooth muscle cell proliferation and viability by suppressing NLRP3-mediated pyroptosis [220]. Therefore, targeting YTHDC1 and inhibiting miR-16–5p represent potential therapeutic strategies for treating thoracic aortic dissection (TAD) [220].

### m^7^G

m^7^G, referring to the methylation of guanine at the seventh nitrogen, is a ubiquitous RNA modification detected in mRNA, tRNA, rRNA, and miRNA, and is known to fulfill critical functions in multiple aspects of RNA metabolism (Fig. 3C). While its presence was first established in the 5’ cap of mRNA, subsequent studies have identified internal m^7^G sites across the 5’-UTR, CDS, and 3’-UTR of mRNAs [221, 222].

#### Dynamic regulation of m^7^G modification

Three major classes of m^7^G methyltransferases have been identified. The METTL1/WDR4 complex serves as a core catalytic unit that introduces m^7^G modification at position 46 (m^7^G46) of tRNA, thereby helping to stabilize its three-dimensional structure [223, 224]. This complex is essential for normal mRNA translation, as well as for the proliferation and differentiation of mouse embryonic stem cells [225]. However, WDR4 mutations are linked to developmental defects [226], and an imbalance in the METTL1/WDR4 complex can promote tumorigenesis [227]. The second complex, RNMT/RAM, is responsible for m^7^G modification at the 5’ cap of mRNA [228, 229]. RAM is required for effective cap methylation both *in vitro* and *in vivo*, and it indirectly supports mRNA expression, translation, and cell viability [229]. Conversely, suppression of RNMT results in decreased cap methylation and loss of cell viability [230]. Lastly, WBSCR22/TRMT112, a functional homolog of the yeast Bud23-Trm112 complex, mediates m^7^G modification in 18S rRNA [231, 232]. In this complex, the small evolutionarily conserved protein TRMT112 acts as a cofactor that assists WBSCR22 in facilitating rRNA m^7^G modification [233].

While m^7^G writers have been well characterized, the enzymes responsible for m^7^G demethylation remain largely unknown, representing a significant gap in our understanding of this modification mechanism.

#### Modulatory effects of m^7^G modification

Under physiological conditions, the m^7^G modification is primarily found at the 5’ end of mRNA, where it forms a typical cap structure (m^7^GpppN). The m^7^G cap is recognized by the eukaryotic translation initiation factor 4E (eIF4E) and the cap-binding complex (CBC), which is composed of CBP80 and CBP20, thereby influencing RNA maturation, nuclear export, and translation [234, 235]. eIF4E is the second protein identified to bind directly to the RNMT methyltransferase domain, following the RNMT cofactor RAM [236]. Additionally, eIF4E can form a ternary complex with the m^7^G cap and RNMT, which facilitates eIF4E’s role in regulating RNA capping and metabolism in the nucleus [236]. Quaking (QKI), a reader of m^7^G modification, specifically recognizes m^7^G-modified internal mRNA. It binds to m^7^G sequences rich in “GA”, facilitating mRNA modification and regulating RNA metabolism, cellular stress responses, and disease development [237, 238]. QKI has three main alternative splicing isoforms, including QKI-5, QKI-6, and QKI-7, each with distinct carboxyl-terminal domains. QKI-5 is primarily located in the nucleus, whereas QKI-6 and QKI-7 are preferentially located in the cytoplasm [239, 240]. By interacting with GTPase-activating protein-binding protein 1 (G3BP1), QKI-6 and QKI-7 transport m^7^G-modified mRNA to stress granules (SG) under stress conditions, thereby regulating the stability and translation of the mRNA [238].

#### The role of m^7^G in diseases

m^7^G modification serves as an important mechanism for epigenetic regulation and is significantly involved in the malignant progression of tumors by influencing various biological processes. A primary effect of m^7^G modification is the promotion of tumor cell proliferation. For instance, METTL1-induced dysregulation of m^7^G tRNA modification has been shown to facilitate tumorigenesis. The METTL1/WDR4 complex is abnormally overexpressed in various tumor types, including lung cancer, ESCC, and head and neck squamous cell carcinoma (HNSCC). This complex enhances mRNA translation efficiency and upregulates the expression of cell cycle-related genes, such as Cyclin D3 (*CCND3*) and Cyclin E1 (*CCNE1*), as well as genes involved in the RPTOR/ULK1/autophagy axis and the PI3K/AKT/mTOR pathway, thereby directly driving tumor cell proliferation [241–243]. Additionally, m^7^G-modified tRNAs can increase the translation efficiency of key oncogenes. For example, they enhance *c-MYC* expression in neuroblastoma, further accelerating tumorigenesis [244]. Moreover, the high expression of METTL1 promotes glioma growth by activating the MAPK signaling pathway [245].

Secondly, m^7^G modification plays a major role in promoting tumor metastasis and invasion. Upregulation of the METTL1/WDR4 complex in NPC induces epithelial–mesenchymal transition (EMT) by activating the Wnt/β-catenin signaling pathway, thereby enhancing tumor metastasis [246]. This finding underscores the vital role of m^7^G modification in regulating tumor cell plasticity and motility. Furthermore, m^7^G modification represents a key mechanism underlying tumor treatment resistance. Beyond promoting metastasis, the METTL1/WDR4 complex in nasopharyngeal carcinoma contributes to resistance against cisplatin and docetaxel through the aforementioned mechanisms [246].

m^7^G regulatory factors display functional diversity across different tumor environments. For example, METTL1 can act as a tumor suppressor in lung cancer by enhancing miRNA processing [242, 247]. In PC, WBSCR22 inhibits the translation of interferon-stimulated gene 15 kDa protein (ISG15) by regulating 18S rRNA m^7^G modification, thereby reducing cell proliferation, invasion, and tumorigenesis [248]. These context-dependent functional differences highlight the complexity of the m^7^G regulatory network.

The m^7^G modification creates a dynamic and adaptable regulatory interface at the transcriptome level. In-depth exploration of its mechanisms not only deepens our understanding of fundamental life processes but also provides novel insights and potential approaches for the diagnosis and treatment of diseases, including tumors.

### m^6^Am

The m^6^Am modification is a prevalent form of adenosine modification that was first identified in animal cells and viral mRNAs in 1975 [249]. This modification arises from methylation at both the *2’*-*O* position of the ribose and the *N*^6^ position of the adenine base. m^6^Am occurs primarily in mRNA and snRNA: in mRNA, it is located at the first nucleotide adjacent to the m^7^G cap structure (cap1), while in snRNA, it is distributed at internal sites, significantly influencing RNA processing and metabolism [250]. Functionally, m^6^Am is closely associated with mRNA stability and translation efficiency (Fig. 3D).

#### Dynamic regulation of m^6^Am modification

The known methyltransferases responsible for m^6^Am formation include phosphorylated CTD interacting factor 1 (PCIF1) and METTL4. PCIF1 has recently been identified as the sole m^6^Am methyltransferase in mammalian mRNA [251], catalyzing m^6^Am methylation on *2’*-*O*-methyladenine at the 5’ ends of mRNAs [252]. It operates through three functional domains: the WW domain interacts with the phosphorylated C-terminal domain (CTD) of RNA polymerase II [253, 254]; the helical domain creates a positively charged groove that recognizes the m^7^G cap structure [253]; and the methyltransferase domain utilizes SAM as a methyl donor to methylate the *N*^6^ position of the first adenosine in mRNA, resulting in the formation of m^6^Am [255, 256]. The second m^6^Am methyltransferase, METTL4, primarily catalyzes the methylation at position 30 of U2 snRNA [257]. On the other hand, FTO is the only known m^6^Am demethylase and specifically demethylates m^6^Am to Am (not m^6^A).

#### Modulatory effects of m^6^Am modification

Additionally, m^6^Am improves mRNA stability by inhibiting the mRNA-decapping enzyme DCP2, reducing microRNA-mediated degradation, and potentially increasing translation efficiency [258]. Recent studies have discovered that premature cleavage factor II (PCF11) is the first reader protein identified to recognize m^6^Am modifications in mRNA that preferentially binds to the region downstream of the transcription start site (TSS) of m^6^Am-modified mRNA [259]. By isolating PCF11 on nascent RNA near the TSS, m^6^Am prevents its binding to the CTD domain (Ser2 phosphorylation site) of RNA polymerase II (RNAP II). This interaction avoids premature dissociation of RNAP II and promotes full-length transcription [259]. When PCF11 expression decreases, the function of m^6^Am is enhanced, leading to increased full-length transcription of ATF3, a transcriptional repressor of the neuroblastoma oncogene *MYCN* [259, 260]. This study not only identifies the reader protein of m^6^Am for the first time, but also proposes a special case of the different relationship between RNA modification and reading protein. It provides a new idea for future disease research.

#### The role of m^6^Am in diseases

The m^6^Am modification, catalyzed by PCIF1, enhances SARS-CoV-2 cellular invasion by stabilizing the mRNA of host invasion factors angiotensin converting enzyme 2 (ACE2) and transmembrane serine protease 2 (TMPRSS2) [261]. Separately, the UL13 protein kinase of the alphaherpesvirus pseudorabies virus (PRV) inhibits the expression of antiviral interferon-stimulated genes (ISGs) by phosphorylating FTO. PRV infection reduces m^6^Am levels in host snRNA and induces phosphorylation of PCIF1, suggesting the critical role of m^6^Am modification in antiviral immune responses [262].

Regarding tumor regulation, PCIF1 exhibits a context-dependent dual function. Overexpression of PCIF1 enhances the proliferation, invasion, and migration of GC and CRC cells, suggesting its carcinogenic properties [263, 264]. Conversely, PCIF1 is frequently downregulated in gliomas and acts as a tumor suppressor [265]. Additionally, PCIF1 regulates the TGF-β and interferon-gamma (IFN-γ) signaling pathways through m^6^Am-dependent mechanisms, thereby influencing the tumor microenvironment and response to immunotherapy [264, 266].

## Pseudouridine

Pseudouridine (Ψ), an isomer of uridine connected to ribose via a carbon-carbon bond, is the first discovered RNA modification [267]. It occurs in nearly all types of RNA, including both coding and non-coding, and is highly conserved across species [268]. This widespread distribution plays a crucial role in regulating gene expression and directing cellular processes during development and disease.

### Dynamic synthesis of Ψ

Ψ is mainly catalyzed by enzymes from the pseudouridine synthases (PUS) family, the TruB family, and Dyskerin pseudouridine synthase 1 (DKC1). Based on their mechanism of action, these enzymes can be broadly categorized into two classes: RNA-independent and RNA-dependent synthases [269]. DKC1 operates in an RNA-dependent manner by forming a complex with H/ACA box small nuclear RNA to catalyze Ψ modification on ribosomal RNA [270]. In contrast, members of the PUS and TruB families, including TRUB1, TRUB2, PUS1, PUSL1, PUS3, PUS7, PUS7L, RPUSD1-4 and PUS10 [271–273], function primarily in an RNA-independent manner, directly converting uridine to Ψ in RNA substrates without the need for a guide RNA [274].

PUS family proteins precisely regulate the Ψ modification in diverse RNA molecules, with their functions influenced by the member-specific functions and cellular localizations [275]. For example, PUS2, structurally homologous to PUS1, specifically modifies the 27 and 28 uridine positions in mitochondrial tRNA, and its dysfunction also leads to mitochondrial impairment [276]. PUS3 primarily targets the 38 and 39 uridine residues in the tRNA anticodon loop; defects in PUS3 not only reduce Ψ levels but also associated with mental retardation [277]. PUS7 recognizes UGUAR-like sequences and helps maintain RNA structural integrity, and its deletion is closely linked to transcriptional instability in myelodysplastic syndrome (MDS) [278, 279]. Furthermore, RPUSD family proteins (RPUSD1-4) cooperatively regulate mitochondrial RNA Ψ modification, and deficiency of RPUSD3 and RPUSD4 impairs translation, resulting in mitochondrial dysfunction [280].

In contrast to the RNA-independent enzymes, DKC1, as the core catalytic component of the RNA-dependent PUS complex, enhances ribosome biosynthesis by modifying the Ψ of rRNA [281]. It also influences the translation of specific mRNAs by regulating ribosome activity, thereby supporting cancer cell proliferation [282]. Beyond its role in rRNA modification, DKC1 contributes to the processing of small nucleolar RNA (snoRNA) and helps stabilize the RNA component of telomerase, and thus maintaining telomere function [283]. DKC1 is overexpressed in multiple malignancies such as CRC, HCC, NBL, and BC, where its upregulation is correlated with enhanced tumor invasiveness and poor prognosis [282, 284].

The synergistic effect of these specific Ψ synthases finely regulates RNA function in different cellular compartments, and their dysfunction is implicated in the development of various diseases, ranging from metabolic disorders to tumors. Despite being discovered over 70 years ago, the action mechanisms and functions of Ψ remain largely unknown. To date, no dedicated Ψ erasers or readers have been identified, and one key direction for future research will be determining whether Ψ is reversible.

### Modulatory effects of Ψ

**Figure 4.**
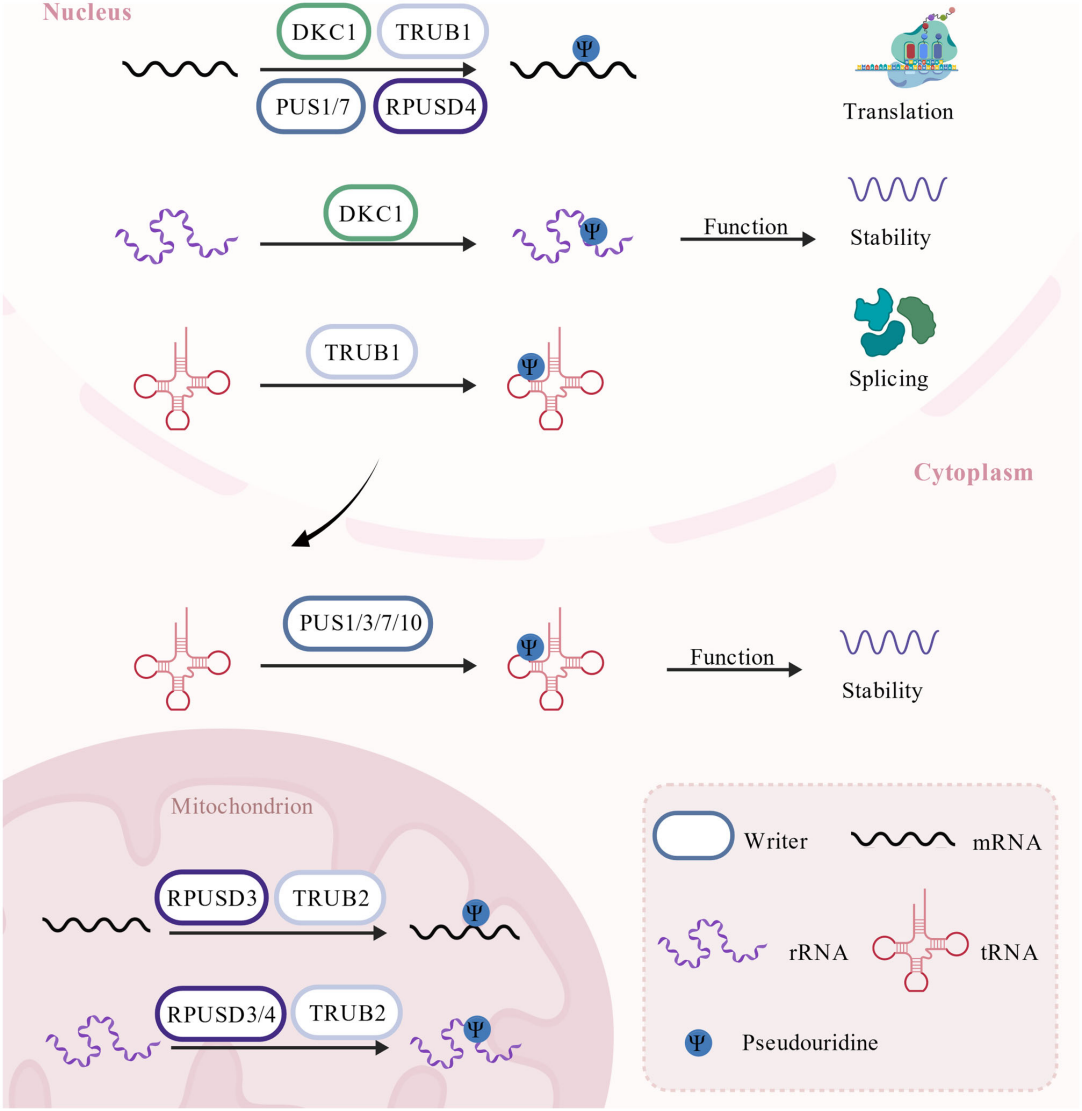
The biosynthesis and functional roles of Ψ modification. Pseudouridine (Ψ) modification occurs in various types of RNA, including mRNA, rRNA, and tRNA, across different cellular compartments. This modification is facilitated by specific writer enzymes, notably DKC1 and PUS family members. To date, no dedicated Ψ erasers or readers have been identified.

Ψ is a pivotal modification of the epigenetic transcriptome, playing a central role in regulating stem cell fate and RNA metabolism through multiple biological processes including translation, stability, and splicing (Fig. 4). In human embryonic stem cells, deficiency of PUS7, a key catalytic enzyme for Ψ, significantly increases both cell volume and protein synthesis rates [285]. Furthermore, it also disrupts mesoderm differentiation, as evidenced by a notable decrease in alpha-smooth muscle actin (α-SMA) positive cells in an embryonic body differentiation model [279].

The regulation of Ψ in RNA metabolism varies across RNA types. In tRNA, Ψ modification occurs not only at position 55 in the T loop but also extends to the D stem and anticodon loop [286], thereby promoting the formation of tRNA-derived fragments (tRFs) in human embryonic stem cells [279]. For rRNA, Ψ enhances the binding affinity of tRNA to the ribosome’s A/P site by improving conformational stability, particularly the structural rigidity of the interaction region between rRNA and tRNA, thereby ensuring translation accuracy [287]. In the case of mRNA, Ψ modification responds dynamically to nutritional status, temperature changes, and disease conditions, playing a role in regulating translation efficiency, accuracy, and stability [288, 289].

The regulation mRNA translation by Ψ occurs through three primary mechanisms. First, at the level of translation efficiency, Ψ can influence the reading of the termination codon [290]. For example, the U-to-Ψ transition in yeast mRNA leads to readthrough of the termination codon, resulting in C-terminally extended protein variants [290]. Additionally, Ψ modifications help mRNA evade PKR-mediated translation inhibition by reducing the affinity of Ψ-modified double-stranded RNA (dsRNA) for the PKR kinase [291]. Second, regarding translation accuracy, Ψ exhibits dual effects. The ΨUU codon can cause valine misincorporation and hinder the ribosome’s ability to proofread mismatched codons, thereby reducing translation fidelity [292, 293]. However, this effect can be dynamically adjusted based on the surrounding sequence and environmental conditions [288, 294]. Lastly, the chemical stability of the C-C glycosidic bond in Ψ significantly prolongs mRNA half-life, which is crucial for *in vitro* mRNA transcription [295].

Furthermore, Ψ is also involved in splicing regulation. For example, Ψ modification in U2 snRNA is essential for its binding to the splicing factor pre-mRNA-processing protein 5 (Prp5) [296]. Blocking this modification site impairs splicing function, while restoring the modification rescues the normal splicing process [296].

### The role of Ψ in diseases

The core regulatory factors of Ψ regulate RNA metabolism, protein synthesis, or cell proliferation/survival in various diseases including tumors, hematological diseases, and mitochondria by mediating RNA Ψ (Table 5).

**Table 5.** Roles of Ψ regulatory factors in diseases.

Diseases	Regulators	Functions
GBM	DKC1, PUS7	DKC1 promotes tumor spread, invasion, and migration [298]; PUS7 promotes cell growth and self-renewal [297].
NSCLC	DKC1	DKC1 promotes proliferation and migration, and inhibits cell apoptosis [305].
HCC	DKC1	DKC1 promotes the migration, proliferation, and invasion of HCC cells [303].
CRC	DKC1, PUS7	DKC1 enhances ribosomal protein expression to promote cancer progression [304]; PUS7 improves the proliferation and invasion of tumor cells [300, 301].
OC	DKC1, PUS7	PUS7 promotes cell proliferation and migration [299].
AML	PUS7	PUS7 promotes the occurrence of leukemia [307].
MLASA	PUS1	PUS1 function is lost and erythropoiesis is reduced [308].
MDS	PUS7	PUS7 dysfunction or deletion promotes malignant transformation [313].

Abbreviations: GBM, glioblastoma; NSCLC, non-small cell lung cancer; HCC, hepatocellular carcinoma; CRC, colorectal cancer; OC, ovarian cancer; AML, acute myeloid leukemia; MLASA, mitochondrial myopathy with lactic acidosis and sideroblastic anemia; MDS, myelodysplastic syndrome.

#### The role of Ψ in tumors

Ψ modification and its key catalytic enzymes, particularly PUS7 and DKC1, play a pivotal role in the malignant progression of various tumors by regulating gene expression and cell metabolism.

##### Regulating proliferative signaling

Many studies indicate the essential function of Ψ in modulating the proliferation of tumor cells. Overexpression of PUS7 directly supports the growth and self-renewal of GBM cells by promoting tRNA Ψ [297]. Conversely, the deletion of DKC1 induces G1 phase cell cycle arrest via modulation of cyclin CDK2 and cyclin Eexpression, effectively inhibiting the proliferation of GBM cells [298]. The mechanism by which PUS7 promotes proliferation has been confirmed in various solid tumors. For instance, PUS7-mediated Ψ modification of *RAP1B* mRNA significantly enhances cell proliferation and migration in OC [299]. Additionally, PUS7 also synergistically drives the growth of CRC by activating the SIRT1-Wnt/β-catenin pathway and the PI3K/AKT/mTOR signaling axis [300, 301].

Beyond directly promoting proliferation, Ψ modification remodels the metabolic state of tumor cells to provide energy support for malignant growth. The PUS7-MYC axis alleviates the metabolic stress associated with rapid proliferation of GBM cells through ATF4-mediated metabolic reprogramming [302]. Similarly, upregulation of PUS1 establishes an energy foundation for HCC cell proliferation and migration by aberrantly activating the mitochondrial respiratory chain and oxidative phosphorylation process [303].

##### Modulating invasion and metastasis

Ψ modification also serves as a key regulator of tumor invasion and metastasis. In CRC, DKC1 promotes tumor angiogenesis and metastasis by directly activating HIF-1α transcription, with its high expression significantly linked to advanced TNM stage and poor prognosis [304]. In another case, lncRNA PCAT1 is markedly upregulated and interacts with DKC1, regulating the proliferation, invasion, and apoptosis of NSCLC cells via the VEGF/AKT/Bcl-2/Caspase-9 pathway [305]. Furthermore, PUS1 upregulation enhances HCC cell migration and invasion by activating multiple signaling pathways, including NF-κB and HIF-1α [303].

##### Impacting cell death

Dysfunction of Ψ-related enzymes contributes to tumor development by impairing apoptosis sensitivity. Genetic variations in PUS10 promote NSCLC cell immortalization and progression by decreasing their sensitivity to tumor necrosis factor-related apoptosis-inducing ligand (TRAIL)-induced apoptosis [306]. Meanwhile, PUS7 dysfunction ultimately fosters leukemia development by increasing protein synthesis and impairing stem cell differentiation [307].

#### The role of Ψ in blood system diseases

Ψ modification provides a core aspect of mitochondrial function maintenance, and its dysregulation is highly correlated with the development of hematological diseases. PUS1 influences cytoplasmic translation by catalyzing pseudouridylation of specific tRNA sites, and its missense mutations are associated with mitochondrial myopathy and sideroblastic anemia (MLASA) [308]. Specifically, when PUS1 modifies position 28 of mitochondrial tRNAs, such as mt-tRNACys and mt-tRNASer (UCN), the molecular stability of these tRNAs is significantly improved [309]. Mitochondrial dysfunction impairs erythropoiesis by disrupting energy metabolism and iron homeostasis, representing a key pathological mechanism underlying PUS1-related blood abnormalities [310]. Further studies have revealed severe mitochondrial impairment, characterized by reduced activity of respiratory chain complexes (especially complex III), decreased oxidative phosphorylation, and increased levels of ROS [311]. Notably, mTOR activation induced by complex III deficiency is closely related to the occurrence of megaloblastic or microcytic anemia [312].

In addition, downregulation of PUS7 expression in MDS promotes a global increase in protein synthesis [279]. MDS patients with low PUS7 expression exhibit a higher risk of leukemia transformation, suggesting the critical role of PUS7 in the pathogenesis of hematological malignancies [313].

### Ψ-related targeted therapy

Ψ-targeting strategies demonstrate considerable promise across multiple therapeutic areas. As diagnostic markers, Ψ levels and alterations in its associated synthases facilitate non-invasive tumor diagnosis. In therapeutic applications, Ψ modification enhances mRNA vaccine development and RNA editing technology by improving mRNA stability and reducing immunogenicity. Additionally, targeting the Ψ-related mTOR pathway offers a novel approach for treating specific diseases. These advances underscore the major potential of Ψ regulation in precision medicine and drug development.

#### As biomarkers for diseases

Abnormally elevated levels of Ψ modification are increasingly recognized as a characteristic of various malignant tumors, showing promise as diagnostic markers. As early as 1983, Salvatore et al. proposed that Ψ in serum could serve as a tumor biomarker [314]. Patients with OSCC exhibit significantly elevated Ψ concentrations in their plasma, urine, and saliva, which may be closely associated with disrupted RNA metabolism in tumor cells [315, 316]. Moreover, mutations and dysfunctions of Ψ synthases are frequently observed in tumors. For instance, aberrant DKC1 leads to excessive Ψ in rRNA, which has been shown to promote tumorigenesis by altering the translation process [317, 318], further supporting the diagnostic relevance of Ψ modification.

In therapeutic applications, Ψ-modified mRNA is particularly valuable due to its low immunogenicity and high stability. This modification effectively diminishes the capacity of *in vitro* transcribed mRNA to activate Toll-like receptor 3 in the endoplasmic reticulum (ER), thereby reducing the immune response [318, 319]. Compared to other RNA modification techniques, Ψ modification minimally affects translation fidelity while preserving RNA targeting efficiency, significantly enhancing treatment safety.

#### As therapeutic targets for diseases

Ψ modification and its derivatives in RNA molecules demonstrate significant potential and complex biological effects in the biomedical field.

Ψ modification is a fundamental technology behind mRNA vaccines, with its derivative, *N*^1^-methylpseudouridine (m^1^Ψ), having been successfully utilized in the Pfizer-BioNTech and Moderna COVID-19 vaccines [319]. This represents a successful clinical application of the modification and has potential value for further research. Studies have shown that m^1^Ψ-modified vaccines can induce high levels of neutralizing antibodies in animal models for Zika virus and influenza virus, providing effective immune protection [320]. This regulation enhances translation efficiency and mRNA stability while reducing immunogenicity, ultimately improving vaccine efficacy. However, it is important to recognize that excessive m^1^Ψ modification may impair the anti-tumor effect by interfering with type I interferon signaling, potentially favoring tumor progression [321, 322].

PUS7 has been identified as a targetable epitranscriptomic regulator of glioblastoma growth, and its high expression is correlated with lower survival rates in tumor patients. Targeting PUS7 suppresses tRNA pseudouridylation and inhibits tumorigenesis [297]. Additionally, dyskerin deficiency resulting from *DKC1* gene mutations is associated with disorders in ER stress, autophagy, and mTOR signaling. This connection highlights potential targets for early intervention in X-linked congenital keratosis (X-DC), such as PERK and mTORC1/2 [323].

## *N* ^4^-acetylcytidine

*N* ^4^-acetylcytidine (ac^4^C) is a conserved RNA modification involving the acetylation of the cytidine at the *N*^4^ position, present across a wide range of RNA molecules in eukaryotes and prokaryotes. This modification is catalyzed by *N*-acetyltransferase 10 (NAT10), which remains the only known writer enzyme for ac^4^C.

### Dynamic synthesis and modulatory effects of ac^4^C

**Figure 5.**
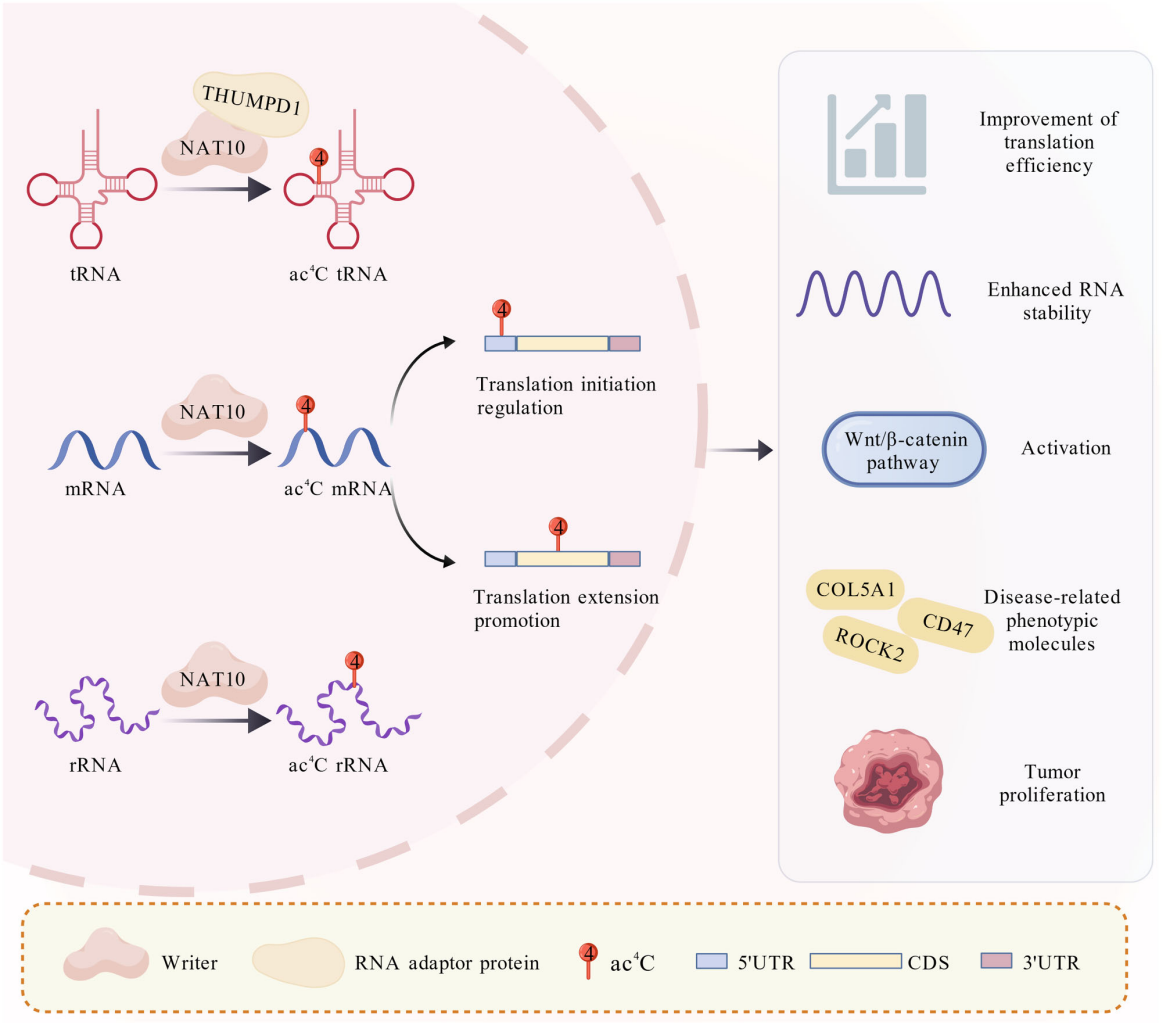
The biosynthesis and functional roles of ac^4^C RNA modification. *N*^4^-acetylcytidine (ac^4^C) modification in RNA is mediated by the writer enzyme NAT10, which utilizes THUMPD1 as a key RNA adaptor protein. NAT10 catalyzes the formation of ac^4^C in tRNA, mRNA, and rRNA. In tRNA, ac^4^C modification is facilitated by THUMPD1. In mRNA, ac^4^C modification in the 5’-UTR regulates translation initiation, while in the coding sequences (CDS), it promotes translation extension. ac^4^C-modified RNA influences various biological processes, including improving translation efficiency, enhancing RNA stability, activating the Wnt/β-catenin pathway, regulating disease-related phenotypic molecules like COL5A1, CD47, and ROCK2, and affecting tumor proliferation.

NAT10, the principal acetyltransferase responsible for ac^4^C modification, is primarily located in the nucleus. Its molecular structure contains functional regions such as the *N*-acetyltransferase domain and the ATP/GTP binding domain [324]. Regarding regulatory mechanisms, ac^4^C can indirectly modulate intracellular signaling pathways by influencing the translation of key regulatory proteins or signaling molecules, thereby altering cell function [324], and supporting complex biological processes and the diversification of cell functions (Fig. 5).

Under physiological conditions, the ac^4^C modification maintains the homeostasis of rRNA and tRNA through the synergistic action of NAT10 and its adaptor THUMP domain-containing protein 1 (THUMPD1) [325, 326]. Under pathological conditions, however, NAT10 overexpression can induce aberrant mRNA acetylation and contribute to disease progression [325]. Studies have shown that the functional role of ac^4^C in mRNA depends on its location: it promotes translation elongation when situated within the CDS, whereas in the 5’-UTR it primarily regulates translation initiation [327]. In specific sequence environments, ac^4^C can also influence the translation initiation process by altering the secondary structure of the Kozak sequence [328, 329].

### The role of ac^4^C in diseases

The ac^4^C modification, mediated by the only known catalytic enzyme NAT10, plays a key role in various disease processes, including infection and autoimmune diseases, and chronic diseases, and especially in tumors (Table 6).

**Table 6.** The representative ac^4^C modification events in human diseases.

Diseases		Regulators	Functions
Tumors	GC	COL5A1	Stabilizing COL5A1 mRNA and promoting EMT and tumor metastasis [330].
		MDM2	HP infection upregulates NAT10, which introduces ac^4^C modification to MDM2 mRNA, stabilizing it and reducing p53 protein levels [340].
		SEPT9	HIF-1α stimulates NAT10 expression and mediates modification on SEPT9 mRNA, increasing SEPT9 translation and protein level [332].
	HNSCC	GLMP	NAT10 promotes tumor metastasis by enhancing ac^4^C-dependent GLMP mRNA stability [331].
	BLC	BCL9L, SOX4, AKT1	NAT10 promotes tumor growth by enhancing its expression via ac^4^C modification [337].
		CLIC3, p21	CLIC3 inhibits NAT10 acetylation, reducing ac^4^C on p21 mRNA [338]; p21 blocks cell cycle, inhibiting tumor proliferation [339].
	BC	ELOVL6, ACSL1/3/4	NAT10 knockdown reduces their stability via ac^4^C, down-regulates fatty acid metabolism genes, lowers intracellular lipids, triglycerides, and cholesterol [334].
		JunB	NAT10 deficiency blocks BC progression by inhibiting JunB-mediated glycolytic pathway [335].
	Osteoarthritis	LDHA, PFKM	NAT10/ac^4^C-YTHDC1/m^6^A axis boosts glycolysis via enhanced enzyme translation, promoting tumor progression [333].
	CRC	FSP1	High NAT10/ac^4^C maintains mRNA stability, up-regulates FSP1, and accelerates tumor growth [336].
		KIF23	NAT10 interacts with its 3’-UTR to enhance ac^4^C/stability, activates the Wnt/β-catenin pathway, and promotes tumor progression [328].
Cardiovascular system diseases	Cardiac remodelling	CD47, ROCK2	NAT10 promotes cardiac remodeling by increasing the ac^4^C modification of CD47 and ROCK2 mRNA, improving its protein expression stability and translation efficiency [342].
Immune system diseases	RA	PTX3	Promotes PTX3 stability and translation to regulate RA FLS invasiveness, linked to synovial invasion/inflammation [345].
	SLE	USP18, GPX1, RGL1	NAT10 expression may correlate with overall ac^4^C level changes in SLE [346].
Nervous system diseases	AD	GRIN1, MAP2, DNAJC6	Abnormal mRNA ac^4^C modification causes hippocampal protein synthesis disorder in early AD mice [347].
	Epilepsy	BDNF	NAT10 expression positively correlates with BDNF; inhibiting the BDNF pathway reverses abnormal NAT10 in the hippocampus [348].
	CPSP	Fn14	NAT10/ac^4^C upregulates Fn14 in hypothalamic neurons, activates NF-κB to mediate central post-stroke pain [349].

Abbreviations: GC, gastric cancer; HNSCC, head and neck squamous cell carcinoma; BLC, bladder cancer; BC, breast cancer; CRC, colorectal cancer; RA, rheumatoid arthritis; SLE, systemic lupus erythematosus; AD, Alzheimer’s disease; CPSP, central poststroke pain.

#### The role of ac^4^C in tumors

NAT10-mediated ac^4^C modification plays a crucial role in the malignant progression of tumors through multiple mechanisms.

##### Modulating invasion and metastasis

The ac^4^C modification significantly impacts tumor invasion and metastasis. NAT10 enhances the stability of collagen type V alpha 1 chain (*COL5A1*) mRNA via the 3’-UTR modification, thereby facilitating EMT and GC cell metastasis [330]. This process is finely regulated by the tumor microenvironment. Similarly, NAT10 promotes metastasis in HNSCC by stabilizing *GLMP* mRNA in an ac^4^C-dependent manner and reshaping the tumor microenvironment through the MAPK/ERK signaling pathway [331]. Additionally, HIF-1α not only initiates NAT10 transcription under hypoxic conditions but is also reinforced by a NAT10/SEPT9/HIF-1α positive feedback loop. This circuit leads to sustained activation of the HIF-1 pathway, which in turn promotes tumor angiogenesis and enhances glycolytic addiction, collectively contributing to resistance against anti-angiogenic therapy [332].

##### Affecting metabolic reprogramming

ac^4^C modification also plays a crucial role in tumor metabolic reprogramming. The recently identified NAT10/ac^4^C-YTHDC1/m^6^A-LDHA/PFKM signaling axis promotes glycolysis in osteosarcoma (OS) cells by a unique mechanism: NAT10-mediated ac^4^C modification suppresses the expression of the m^6^A reader YTHDC1, which in turn reduces the m^6^A methylation and stability of key glycolytic enzymes LDHA and PFKM mRNAs, ultimately enhancing glycolytic flux and supporting tumor progression [333]. In BC, NAT10 deletion decreases the mRNA stability of key fatty acid metabolism genes *ELOVL6* and *ACSL1* [328, 334], while the ac^4^C-JunB-LDHA axis activates the glycolytic pathway and contributes to an immunosuppressive microenvironment [335].

##### Regulating proliferative signaling

ac^4^C modification directly modulates tumor cell proliferation and survival. NAT10 facilitates CRC growth through multiple mechanisms. For instance, it can stabilize the mRNA of the ferroptosis suppressor gene *FSP1* by ac^4^C acetylation. Alternatively, it activates the Wnt/β-catenin pathway through interaction with the 3’-UTR of *KIF23* mRNA [328, 336]. In BLC, NAT10 promotes tumor growth by upregulating key oncogenes such as B-cell CLL/lymphoma 9-like (*BCL9L*), *SOX4*, and *AKT1* through ac^4^C modification [337]. In contrast, chloride intracellular channel 3 (CLIC3) inhibits tumor proliferation by inhibiting NAT10 acetylation and reducing the ac^4^C level on *p21* mRNA [338, 339], revealing an internal balance mechanism within this pathway. Moreover, *Helicobacter pylori* (*H. pylori*) infection stabilizes *MDM2* mRNA and decreases p53 protein levels by upregulating NAT10 expression, creating conditions that favor GC [340].

In summary, NAT10-driven ac^4^C modification constitutes a complex regulatory network that coordinates tumor cell invasion, metabolism, and proliferation. These insights advance our understanding of tumorigenesis and provide a multifaceted theoretical foundation for developing new combination therapy strategies.

#### The role of ac^4^C in other diseases

NAT10 catalyzes the ac^4^C modification, a highly conserved RNA modification mechanism that regulates physiological and pathological processes in cardiovascular, immune, and neurological diseases.

##### Cardiovascular diseases

Within the cardiovascular system, NAT10-mediated ac^4^C modification influences cardiac remodeling by regulating the expression of key genes. Specifically, NAT10 enhances the translation efficiency and stability of cluster of differentiation 47 (CD47) and Rho-associated coiled-coil containing protein kinase (ROCK2) mRNAs through ac^4^C modification [341, 342]. Conversely, in NAT10 knockout hearts, the translation efficiency of the histone methyltransferase lysine methyltransferase 5a (Kmt5a) is significantly impaired, leading to the disruption of normal heart function [343].

##### Immune and inflammatory diseases

In immune and inflammatory diseases, NAT10-mediated ac^4^C modification plays a central role in the inflammatory signal transduction. NAT10 knockout significantly reduces the production of inflammatory factors, whereas its overexpression exerts the opposite effect. For instance, in a periodontitis model, NAT10 expression is upregulated during osteoclast formation, and *in vitro* experiments confirm its direct role in promoting osteoclast differentiation. Accordingly, the administration of the NAT10 inhibitor Remodelin effectively reduces inflammatory bone loss *in vivo* [344]. NAT10-mediated ac^4^C modification also contributes significantly to autoimmune diseases. In patients with rheumatoid arthritis, this modification enhances the invasive capacity of rheumatoid synovial cells and exacerbates the local inflammatory response in the joints, further promoting joint destruction [345]. In systemic lupus erythematosus, abnormal NAT10 expression shows a significant correlation with global ac^4^C levels, suggesting its involvement in the pathogenesis of autoimmune disorders through the regulation of ac^4^C modification in downstream target gene transcripts [346]. Findings from these studies further validate the clinical potential of targeting ac^4^C-related pathways in autoimmune disease therapy.

##### Neurological diseases

In the context of neurological diseases, dysregulated protein synthesis in the hippocampus during early AD is closely linked to aberrant ac^4^C modification [347]. In epilepsy models, the brain-derived neurotrophic factor (BDNF) pathway positively regulates NAT10 expression [348]. Additionally, NAT10 in the thalamus upregulates fibroblast growth factor-inducible 14 (Fn14) expression through ac^4^C modification, activating the NF-κB signaling pathway and promoting the occurrence and progression of central poststroke pain (CPSP) following thalamic hemorrhage [349]. These observations suggest that targeted inhibition of NAT10 and its mediated ac^4^C modification may offer a novel therapeutic strategy for neurological diseases.

### ac^4^C-related targeted therapy

Given the pivotal role of ac^4^C modification across various diseases, it presents new diagnostic and therapeutic opportunities for cancer, heart disease, immune-inflammatory diseases, and neurological disorders.

NAT10, frequently upregulated in various tumors, mediates mRNA ac^4^C modification and plays a vital role in tumor proliferation, drug resistance, DNA damage repair, and cell metabolism, making it a promising target for anticancer therapy [334, 336, 337]. Studies indicate that ac^4^C modification strongly correlates with lymph node metastasis in HNSCC, and elevated NAT10 expression may predict tumor susceptibility and unfavorable prognosis [331]. Moreover, combining a NAT10 inhibitor with gefitinib significantly delays the progression of esophageal cancer, offering a viable strategy to overcome targeted drug resistance [350].

During cardiac remodeling, ac^4^C modification participates in disease pathogenesis, and the NAT10 inhibitor Remodelin has been shown to prevent cardiac insufficiency by inhibiting cardiac fibrosis, hypertrophy, and inflammatory responses [342]. In immune-inflammatory contexts, Remodelin also reduces macrophage infiltration and absorption, highlighting its broad therapeutic potential [344, 351].

Studies have shown that NAT10 protects against neurological diseases. For example, an early-stage AD mouse model exhibits abnormal modifications of ac^4^C, which affect mRNA stability and protein expression and may contribute to disease initiation and progression. Thus, ac^4^C shows the promise as a biomarker for early detection and prevention [347]. Furthermore, the transcription factor USF1 enhances NAT10 expression by binding to its promoter and mediates neuropathic pain through ac^4^C modification of target mRNAs such as Syt9 [352]. As a potential target for treating neuropathic pain, NAT10 provides a molecular basis for developing targeted analgesic strategies.

## A-to-I RNA editing

A-to-I RNA editing is a post-transcriptional modification process catalyzed by adenosine deaminases acting on RNA (ADAR) enzymes, which deaminate adenosine (A) to inosine (I) in dsRNAs. This conversion fundamentally alters the base-pairing property of RNA, as inosine (I) is recognized as guanosine (G) during translation and RNA-RNA interactions. As a result, A-to-I RNA editing can lead to changes in protein-coding sequences, influence splicing patterns, and modulate non-coding RNA functions [353–355].

### Catalytic mechanism of A-to-I RNA editing

In humans, three ADAR family members have been identified, including ADAR1, ADAR2, and ADAR3. However, only ADAR1 and ADAR2 exhibit editing activity [356, 357]. ADAR1 is widely expressed across various tissues and possesses two isoforms, p110 and p150, with the latter primarily targeting repetitive RNA sites [358, 359]. The HECT-type E3 ubiquitin ligase SMURF2 acts as a key regulator of ADAR1 p110 by stabilizing the protein and enhancing its editing function via E3 ligase-dependent ubiquitination at lysine 744 (K744) [360]. In contrast, ADAR2 is predominantly expressed in the nucleus of the CNS, where its editing activity is primarily focused on non-coding RNA regions [361, 362]. Although ADAR1 and ADAR2 share 262 co-editing sites [362, 363], they are functionally non-redundant *in vivo* [364]. Furthermore, inosine is recognized as guanosine by the cellular translation machinery during protein synthesis, leading to the alternative designation of A-to-I editing as A-to-G editing [365]. This editing process partially compensates for genomic G-to-A mutations by inducing A-to-G changes at the RNA level [362, 366]. Importantly, non-synonymous A-to-I RNA editing promotes the retention of predicted deleterious G-to-A missense mutations within human populations [367].

### Regulatory networks and physiological functions of A-to-I RNA editing

**Figure 6.**
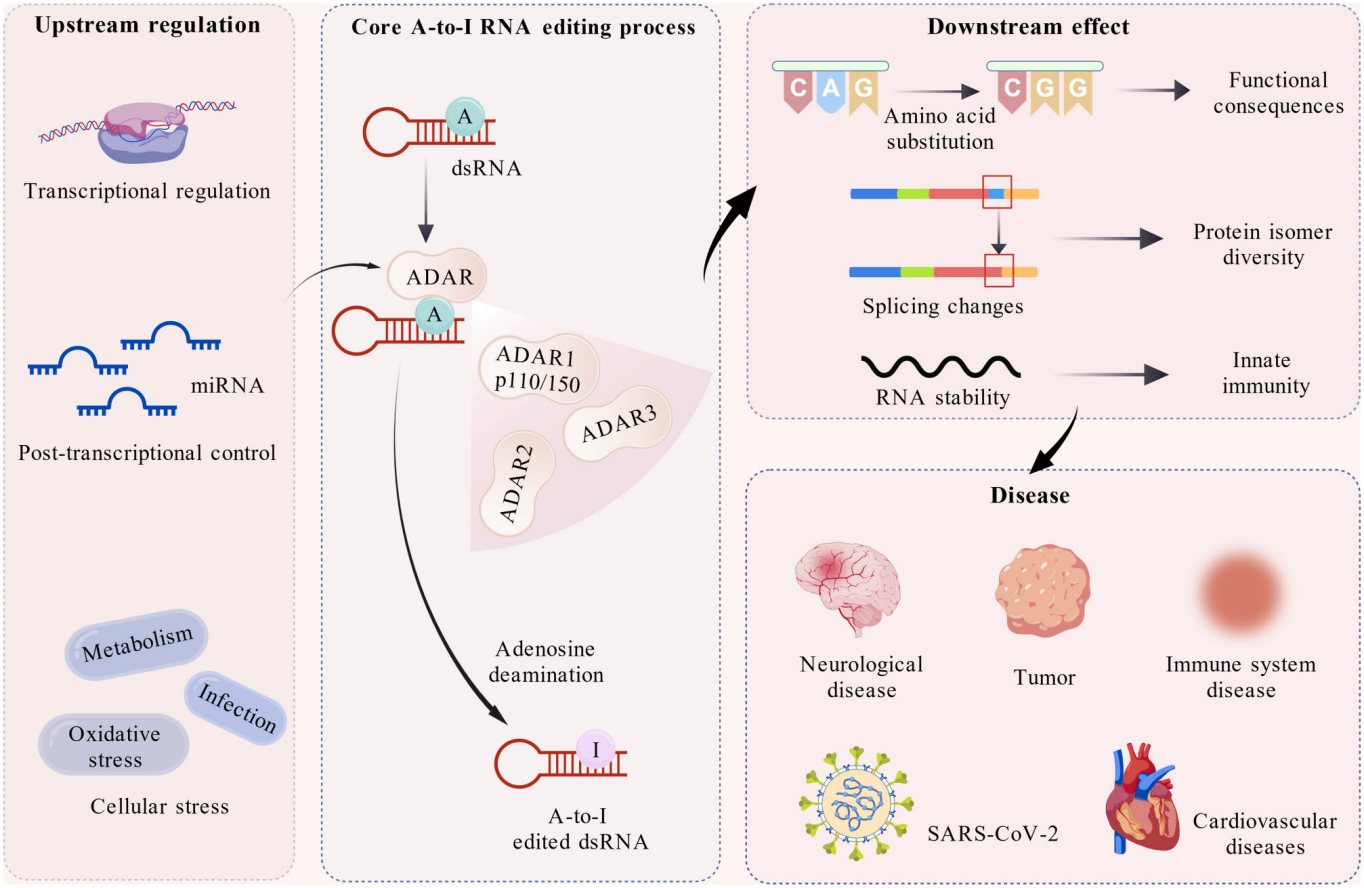
The mechanism of A-to-I RNA editing. The regulatory network of adenosine-to-inosine (A-to-I) RNA editing involves upstream regulation, a core editing process, downstream effects, and disease implications. Upstream regulation encompasses transcriptional control, post-transcriptional regulation (e.g. miRNA), and cellular stress responses (e.g. metabolites, infections, oxidative stress). During the core A-to-I RNA editing process, ADAR family members (ADAR1, ADAR2, ADAR3) catalyze the deamination of adenosine (A) to inosine (I) in dsRNA of pre-mRNA. This editing induces downstream functional consequences, including amino acid substitution, protein isomer diversity (via splicing changes), and impacts on RNA stability and innate immunity. This mechanism is also associated with various diseases spanning neurological diseases, tumors, immune system diseases, SARS-CoV-2 infection, and cardiovascular diseases.

ADAR-mediated A-to-I RNA editing is a fundamental regulatory mechanism involved in various biological processes, including transcript stability, splicing, translation, and miRNA silencing (Fig. 6).

#### Regulatory networks of A-to-I RNA editing

Studies indicate that transcriptional activity and precursor mRNA processing finely tune A-to-I RNA editing levels. For example, activation of the transcription factor MYC elevates A-to-I modification levels, while lower precursor mRNA synthesis rates similarly enhance the modification extent [368]. This variation in modification levels primarily results from the alternative splicing of modified precursor mRNAs and the regulation of the transcription process [368].

RNA-binding proteins contribute significantly to cell-type-specific regulation of ADAR expression and substrate interactions [369]. Importantly, the editing activity of ADARs can be influenced by specific protein interactions. Death-associated protein 3 (DAP3), for instance, directly binds to the ADAR2 deaminase domain, disrupting its interaction with target transcripts and inhibiting editing activity, resulting in cancer cells escaping from editing-mediated tumor suppression [370]. In contrast, ADAR1 deletion decreases editing of interferon-induced RNAs, leading to recognition and accumulation of double-stranded RNA by sensors like PKR and MDA5. This process activates the anti-tumor immune response and tumor inhibition mechanisms, ultimately altering the tumor microenvironment [371].

In early human embryogenesis, genome-wide RNA modification undergoes dynamic changes. A-to-I RNA editing levels decrease steadily from the stable state observed at the 4-cell stage, persisting through the 8-cell and morula stages [372]. Notably, ADAR1 deficiency triggers melanoma differentiation-associated gene 5 (MDA5) pathway-dependent cell death, and even blocking the apoptotic pathway fails to prevent the death of ADAR1-deficient embryos [373].

A-to-I RNA editing during brain development shows unique spatiotemporal specificity and genetic regulation. Multi-dataset analyses indicate that the overall editing level of Alu elements significantly increases with brain maturity, a trend that is evolutionarily conserved in mammals, while editing in the 3’-UTR regions can affect miRNA binding efficiency [374]. However, RNA hyperediting in the aging brain may enhance RNA structure stability, and certain cis-edited quantitative trait loci play differential regulatory roles before and after birth [374]. Editing levels of A-to-I in brain tissue increase after death, potentially linked to the sustained ADAR1 and ADARB1 expression under inflammatory and hypoxic conditions that occur post-mortem [375]. Moreover, there is a remote coupling between the A-to-I RNA editing (Q/R editing) of exon 20 and the alternative splicing of exon 4 in transmembrane protein 63B (Tmem63b) precursor mRNA. This synergistic mechanism regulates the Ca^2+^ permeability and osmosensitivity of channel proteins, and thereby participating in its functional role within the brain [376].

#### Physiological functions of A-to-I RNA editing

The functional consequences of A-to-I modification are multifaceted. Inosine exhibits distinct base pairing characteristics compared to adenosine, inducing structural rearrangements through sequence alteration, which in turn affects mRNA translation, pre-mRNA splicing, and miRNA silencing efficiency [377]. A-to-I editing occurs predominantly in primate-specific Alu elements, which can form local double-stranded RNA structures [378, 379]. However, due to the relatively weak binding force between inosine and uracil, RNA editing may promote RNA duplex unwinding, thereby sensitizing substrate RNAs to single-stranded-specific RNases [380].

Besides its core regulatory role, A-to-I RNA editing plays a significant part in regulating specific physiological processes. For example, a deficiency in folate (FA) inhibits ADAR3-mediated A-to-I deamination, resulting in the accumulation of endogenous dsRNA within the cell. This process triggers a host immune response, increases 2’-5’-oligoadenylate synthetases 2 (OAS2) expression, and ultimately suppresses viral replication [381]. This editing is essential for maintaining normal vasoconstriction and blood pressure, as it inhibits the RhoA/ROCK and PLC/PKC signaling pathways by regulating the membrane localization of p190RhoGAP [382]. Furthermore, under ischemic conditions, several A-to-I RNA-edited miRNAs are highly expressed and specifically regulated by ADAR1 and ADAR2, indicating the effect of A-to-I editing in the process of angiogenesis [383].

### The role of A-to-I RNA editing in diseases

A-to-I RNA editing mainly shows differential changes in tumors (Table 7), the nervous system, the immune system, the cardiovascular system, and other diseases (Table 8), and participates in the regulation of corresponding pathological processes.

**Table 7.** Representative A-to-I RNA editing events in tumors.

Tumors	Regulators	Functions
NSCLC	CYP1A1	I462V editing induces HO-1 expression via PI3K/Akt activation, enhances CYP1A1-HO-1 interaction, and promotes HO-1 nuclear translocation to resist oxidative stress [385].
	SNHG3	SNHG3 super-editing enhances binding to SSRP1, activates transcriptional elongation, up-regulates fatty acid metabolism and ferroptosis-related genes, leading to docetaxel resistance [388].
	miR-411–5p	Edited form directly targets MET, inhibits MAPK/ERK pathway and AP1 activity, reversing TKI resistance [386].
CRC	AZIN1	Editing endows cancer stemness, up-regulates IL-8 to promote angiogenesis; delays c-Myc degradation via OAZ2-mediated non-ubiquitin pathway, increasing IL-8 transcription [391, 392].
	COPA	I164V editing induces endoplasmic reticulum stress, promotes ATF6/XBP1/ATF4 nuclear translocation, activates MALAT1/MET/ZEB1 expression, leading to invasion and metastasis [389].
HCC	miR-3144–3p	ADAR1-mediated editing weakens inhibition on MSI2, activates MET signal; inhibits SLC38A4, enhancing malignant phenotype [394].
	KPC1	A-to-I overediting of KPC1 residue 8 (p.M8V), reduces KPC1-NF-κB1 p105 affinity, decreases p105 ubiquitination, and activates NF-κB signaling [395].
Melanoma	miR-378a-3p	Editing in non-metastatic cells binds to PARVA 3’-UTR, inhibits PARVA expression, and prevents malignant phenotype [390].
GBM	PHKA2	ADAR2-mediated editing regulates PHKA2; PHKA2 phosphorylates EBF1, reducing its stability, forming SNORD113-3/ADAR2/PHKA2/EBF1 pathway to control glycolipid metabolism and cell growth [387].
PTC	CDK13	Q103R editing (ADAR1-mediated) enhances CDK13’s ability to promote cell proliferation, survival, and invasion, altering nuclear localization [428].
	miR-200b	ADAR1 edits miR-200b seed region, weakens binding to ZEB1, relieving ZEB1 inhibition; RAS regulates ADAR1 via PI3K pathway [397].

Abbreviations: NSCLC, non-small cell lung cancer; CRC, colorectal cancer; HCC, hepatocellular carcinoma; GBM, glioblastoma; PTC, papillary thyroid carcinoma.

**Table 8.** Representative A-to-I RNA editing events in other diseases.

Diseases		Regulators	Functions
Nervous system diseases	AD	GluA2 (AMPA receptor subunit)	Decreased editing in the hippocampus/temporal lobe/frontal lobe affects receptor function; unmodified Alu RNA triggers an innate immune response via extracellular vesicles [401, 406].
	PD	miR-497–5p	The overexpression of edited miR-497–5p may aggravate neurodegenerative diseases by inhibiting genes such as OPA1 [403].
	ALS	circGRIA2	ADAR2 activity changes affect circGRIA2 editing; ADAR2 loss leads to GluR2 unmodified-mediated motor neuron death [406].
Immune system diseases	SSc	ADAR1 p150, CTSS	ADAR1 up-regulation increases CTSS 3’-UTR Alu editing, enhancing CTSS stability; ADAR1 silencing reduces CTSS expression [413].
	IBD	Alu RNAs	Decreased Alu editing in white blood cells; ADAR1 down-regulation in UC intestinal epithelium enhances TNF-α/IFN-β release and PANoptosis [415].
Infectious diseases	SARS-CoV-2 infection	ADAR1 p150	Up-regulated in cytotoxic CD8^+^ T cells, maintaining T cell homeostasis; reduced lung Alu editing activates the IRF/NF-κB pathway [420].
		Multiple host RNAs	Transiently increased editing during disease; vaccine-induced editing regulates APOL6/IFI30/GBP1, modulating immunity [432].
Cardiovascular system diseases	Atherosclerosis	NEAT1	ADAR1 edits NEAT1 3’-end Alu region, promoting AUF1 binding and NEAT1 stability, enhancing endothelial cell pro-inflammatory response [422].
	HF	Alu elements	ADAR2 proteasomal degradation reduces Alu editing, promoting reverse Alu-mediated circRNA generation [425].
	HPH	circCDK17	ADAR1 edits circCDK17, inhibits m1A modification, reduces the stability of circCDK17, and reduces the inhibition of PASMC proliferation [426].

Abbreviations: AD, Alzheimer’s disease; PD, Parkinson’s disease; ALS, amyotrophic lateral sclerosis; SSc, systemic sclerosis; IBD, inflammatory bowel disease; HF, heart failure; HPH, hypoxic pulmonary hypertension.

#### The role of A-to-I RNA editing in tumors

A-to-I RNA editing constitutes a complex regulatory network that integrates key biological processes in cancer, including therapeutic resistance, metabolic reprogramming, metastasis and invasion, and the acquisition of stem cell-like properties. This network not only enhances our understanding of tumorigenesis mechanisms but also provides a multifaceted theoretical foundation for developing new treatment strategies. Although no definitive cancer-specific editing sites have been identified; however, A-to-I editing consistently influences editing frequency, gene expression profiles, protein function, splicing patterns, and miRNA-mediated regulation of tumor-related genes [384].

##### Adjusting drug resistance

A-to-I RNA editing contributes critically to tumor treatment resistance. Specifically, A-to-I RNA editing of cytochrome P450 family 1 subfamily A member 1 (CYP1A1) enhances the nuclear translocation of heme oxygenase-1(HO-1) by activating the PI3K/Akt pathway, thereby increasing the resistance of NSCLC cells to oxidative stress [385]. Additionally, the editing of miR-411–5p alters its targeting properties, further promoting drug resistance by inhibiting the MAPK/ERK pathway and activator protein 1 (AP1) activity by targeting cellular-mesenchymal epithelial transition factor (c-MET) [386].

##### Affecting metabolic reprogramming

A-to-I RNA editing also regulates malignant progression by remodeling tumor metabolism. SNORD113-3 enhances the expression of ADAR2, which promotes the editing of PHKA2 and subsequently regulates glucose and lipid metabolism as well as glioblastoma progression via the PHKA2/EBF1 axis [387]. A similar metabolic reprogramming occurs in NSCLC, where excessive editing of SNHG3 increases the binding affinity of the chromatin remodeling factor SSRP1, upregulating the expression of genes related to fatty acid metabolism and ferroptosis. This process ultimately enhances fatty acid oxidation, confers resistance to ferroptosis, and promotes cancer cell metastasis [388].

##### Modulating invasion and metastasis

A-to-I RNA editing is a key mechanism affecting tumor invasion and metastasis. In CRC, the A-to-I RNA-edited COPA activates the expression of pro-invasive genes such as *MALAT1* and *MET* by inducing endoplasmic reticulum stress [389]. Reduced ADAR1 expression in melanoma leads to the accumulation of unedited miR-378a-3p, which loses its ability to suppress PARVA, thereby promoting a malignant phenotype [390]. Notably, A-to-I editing can also confer stem-like traits and stimulate angiogenesis. In digestive system tumors, editing of AZIN1 delays c-MYC degradation and upregulates IL-8, endowing cancer cells with stemness and promoting angiogenesis [391, 392]. These features are closely associated with therapy resistance and tumor recurrence.

Remarkably, the A-to-I RNA editing regulatory network is highly context-dependent. HCC exhibits a notable imbalance in A-to-I RNA editing, primarily characterized by overexpression of ADAR1 and down-regulation of ADAR2 [393]. In this setting, ADAR1 functions as an oncogene, whereas ADAR2 acts as a tumor suppressor [393]. For instance, ADAR1 alleviates miR-3144–3p-mediated suppression of the oncogene *MSI2*, thus activating the MET signaling pathway [394]. In intrahepatic cholangiocarcinoma (iCCA), ADAR1 enhances carcinogenic signals by mediating RNA editing of KPC1, which reduces the binding affinity of NF-κB1 p105 [395]. Conversely, ADARB2, a newly identified tumor suppressor, inhibits the viability and invasion of papillary thyroid carcinoma cells [396]. This functional diversity underscores the complexity of the A-to-I RNA editing network. Moreover, studies indicate that 8-aza can reduce tumor malignancy by inhibiting RAS-mediated regulation of ADAR1 via the PI3K pathway [397], suggesting a potential direction for therapies targeting A-to-I RNA editing.

#### The role of A-to-I RNA editing in neurological diseases

A-to-I RNA editing plays a significant role in the epigenetic regulation of nervous system diseases.

##### Brain injuries

Following TBI, the expression of ADAR1 is notably downregulated, while circHtra1 remains up-regulated. circHtra1 regulates growth factor receptor-bound protein 10 (GRB10) expression by adsorbing miR-3960, which promotes neuronal apoptosis and worsens neuronal cell loss after brain injury [398]. Interestingly, in the sepsis-associated encephalopathy (SAE) model, ADAR1 p150 isoform exerts neuroprotective effects by inhibiting Z-DNA/RNA binding protein 1 (ZBP1) dependent neuronal necroptosis through A-to-I RNA editing [399].

##### Neurodegenerative diseases

Aberrant A-to-I RNA editing levels are commonly observed in neurodegenerative diseases, with regulatory mechanisms involving variations in RNA-binding proteins such as eIF4A3, U2AF2, and NOP58. These variations result in significant molecular phenotypic changes, impacting protein function, gene expression, and RNA splicing [400]. In AD, the overall editing levels in the temporal and frontal lobes exhibit a downward trend, directly affecting the expression of key proteins such as the AMPA receptor subunit GluA2 [401]. This decline is particularly pronounced in carriers of the apolipoprotein Eε4 allele [401]. The risk of developing PD is strongly associated with editing-site mutations in genes such as *NCOR1, KANSL1*, and *BST1* [402]. Additionally, overexpression of the edited miR-497–5p may worsen neurodegenerative conditions by inhibiting genes like *OPA1* [403]. Moreover, α-synuclein oligomers positively activate Toll-like receptor (TLR) and interferon pathways in astrocytes, leading to the upregulation of ADAR1 expression and the modulation of neuroinflammation through editing events [404]. In the nuclei of neurons affected by PD and Lewy body dementia, inclusions formed by NONO/SFPQ protein and A-to-I edited RNA may enhance editing in the Alu regions by reducing ADAR3 expression [405]. In ALS, disease progression is significantly influenced by altered ADAR2 activity. Functional alterations in ADAR2 enhance the editing efficiency of key RNAs, while loss of ADAR2 function can result in unedited GluR2-mediated motor neuron death [406]. In models of *C9orf72* gene-related pathology, abnormal localization of ADAR2 leads to a broad array of A-to-I RNA editing mutations, impacting critical pathways such as the integrated stress response (ISR) and protein translation [407, 408].

#### The role of A-to-I RNA editing in immune system diseases

A-to-I RNA editing serves as a key mechanism for immune regulation. ADAR1 prevents aberrant activation of type I interferon responses by editing specific double-stranded RNAs, thereby inhibiting sustained signaling through nucleic acid sensors such as MDA5, PKR, and ZBP1 [409, 410]. During induced pluripotent stem cell reprogramming, loss of ADAR1 triggers abnormal innate immune response via the MDA5 sensor, disrupting the mesenchymal-epithelial transition [411]. Notably, interferon stimulation enhances the translation efficiency of ADAR1 p150 through the m^6^A reader YTHDF1, creating a positive feedback loop that further amplifies the immune-related editing effects [412].

Differential A-to-I RNA editing patterns are observed in autoimmune diseases. In systemic sclerosis (SSc), the expression of ADAR1 p150 and cathepsin S (CTSS) is elevated in both skin and peripheral blood, accompanied by significantly increased editing of an Alu element in the 3’-UTR of the *CTSS* gene [413]. Elevated ADAR1 p150-mediated editing promotes inflammatory gene expression in rheumatoid arthritis synovial tissue, which is significantly improved after treatment [414]. By contrast, patients with inflammatory bowel disease (IBD) show decreased Alu RNA editing levels, which negatively correlate with increased expression of interferon-stimulated genes (*ISGs*) [415].

A-to-I RNA editing plays multiple regulatory roles in infection and immunity, influencing bacterial infections, antiviral responses, and vaccine efficacy. This editing process has been shown to impact the pathogenicity of *Klebsiella pneumoniae* by regulating its virulence genes during bacterial infections [416]. ADAR1 and ADAR2 exhibit distinct functions during viral infections. ADAR1 deficiency enhances type I interferon production by activating the RLRs-MAVS pathway, thereby increasing resistance to HPV16 and HIV-1 [417, 418]. Conversely, during the early stages of Borna disease virus (BoDV) infection, ADAR2 facilitates viral RNA camouflage by introducing A-to-G mutations, enhancing infection efficiency [419]. In the context of SARS-CoV-2 infection, high expression of ADAR1 p150 in CD8^+^ T cells correlates with disease severity and plays a role in maintaining T cell homeostasis [420].

#### The role of A-to-I RNA editing in cardiovascular diseases

A-to-I RNA editing modulates pathological processes in various cardiovascular diseases. In conditions such as atherosclerosis and cardiomyopathy, elevated editing levels and the persistent upregulation of the inflammatory gene *IGFBP7* highlight the functional importance of A-to-I editing in these pathological mechanisms [421].

Atherosclerosis development is influenced by multiple A-to-I RNA editing events. For instance, increased expression of the pro-inflammatory long non-coding RNA NEAT1 intensifies the vascular endothelial cell inflammatory response induced by TNF-α. ADAR1 further exacerbates inflammation by catalyzing A-to-I RNA editing of the Alu element at the 3’-end of NEAT1, which facilitates the binding of the RNA-binding protein AUF1 and helps stabilize NEAT1 transcripts [422]. Concurrently, editing of Alu inverted repeats in the 3’-UTR region of *CTSS* mRNA in endothelial cells recruits the HuR protein, enhancing mRNA stability under hypoxic or inflammatory conditions and further accelerating disease progression [380]. Beyond endothelial cell-related mechanisms, the expression of ADAR1 in smooth muscle cells is implicated in atherosclerosis-related immune responses by regulating the activation of the double-stranded RNA sensor MDA5 [423].

In children with congenital heart disease, blood samples show a significant increase in the editing rate of the Alu element region within intron 9 of the mediator complex subunit 13 (*MED13*) gene, accompanied by decreased ADAR2 expression, suggesting a potential link between aberrant editing and congenital heart disease [424]. ADAR2-mediated A-to-I RNA editing inhibits the formation of Alu element dsRNA structures, while reduced ADAR2 levels lead to increased circRNA production, indicating a causal role for ADAR2 downregulation in the elevated circRNA levels observed in heart failure patients [425]. Conversely, in hypoxic pulmonary hypertension (HPH), ADAR1 expression is significantly upregulated in lung tissue, where it inhibits m^1^A modification and reduces the stability of circRNA. This mechanism diminishes the inhibitory effect of circCDK17 on the proliferation of pulmonary artery smooth muscle cells (PASMCs), thereby accelerating disease progression [426].

### A-to-I RNA editing-related targeted therapy

A-to-I RNA editing holds considerable value in disease diagnosis and treatment. For diagnosis, COPA A-to-I editing in CRC can predict immunotherapy response, while AZIN1 editing influences tumor angiogenesis via IL-8 regulation [389, 392]; elevated AZIN1 editing in GC serves as an independent risk factor for prognosis and lymph node metastasis [427]. Similarly, the editing imbalance resulting from ADAR1 overexpression and ADAR2 downregulation in HCC indicates poor prognosis [393]. Together, these findings establish A-to-I RNA editing as a major cross-disease diagnostic marker.

ADAR1 and its associated RNA editing process have emerged as promising therapeutic targets in thyroid cancer, HCC, T-ALL, and triple-negative breast cancer [428–431]. In lung cancer, CYP1A1 A-to-I RNA editing drives carcinogenesis through the CYP1A1-HO-1-PI3K/Akt axis [385], while edited miR-411–5p represents a potential target for reversing TKI resistance by targeting MET [386]. Additionally, the ADAR1-miR-3144–3p-MSI2/SLC38A4 axis and the ADAR1-KPC1-NF-κB axis constitute key therapeutic pathways in HCC [394, 395]. Based on the protective effect of ADAR1 on UC and the discovery that specific AluRNA subsets activate the IRF/NF-κB pathway, researchers have identified innovative targeting strategies for tumor immunotherapy and vaccine development [415].

In cardiovascular diseases, A-to-I RNA editing regulates angiogenesis-related miRNA expression, inflammation-associated lncRNA stability, and the circRNA-m^1^A axis, offering novel insights into therapeutic approaches [383, 422, 426]. Moreover, the combined effects of FA metabolism and the ADAR3/endogenous dsRNA/OAS axis may represent a novel targeted therapy strategy for RNA virus infections [381]. A-to-I RNA editing also plays a unique role in vaccine immunization. For instance, the COVID-19 vaccine induces dynamic A-to-I RNA editing in blood, modulating host immune response through epigenetic modifications of the *APOL6* gene and dose-dependent missense editing of genes such as *IFI30* and *GBP1* [432].

## C-to-U RNA editing

C-to-U RNA editing serves as an important regulatory mechanism for biodiversity, primarily mediated by enzymes of the apolipoprotein B mRNA editing catalytic polypeptide (APOBEC) family. These enzymes catalyze the hydrolytic deamination of cytosine (C) to uracil (U) in RNA substrates.

### Catalytic mechanism of C-to-U RNA editing

Among these enzymes that catalyze C-to-U RNA editing, APOBEC3A functions as a cytidine deaminase that acts on RNA substrates and contributes to innate immunity [433]. APOBEC3G, an endogenous RNA editing enzyme, is highly expressed in cytotoxic lymphocytes and has been identified in primary natural killer cells and lymphoma cell lines [434]. These enzymes were initially recognized for editing virus-derived single-stranded DNA, and their RNA editing capability was not established until 2015 [435, 436]. Further analyses have revealed that a subset of non-synonymous DNA single nucleotide polymorphism sites, which convert RNA cytosine to uracil, are either targets of RNA editing by APOBEC3A/G or result from transient RNA editing [437].

### Regulatory networks and physiological functions of C-to-U RNA editing

**Figure 7.**
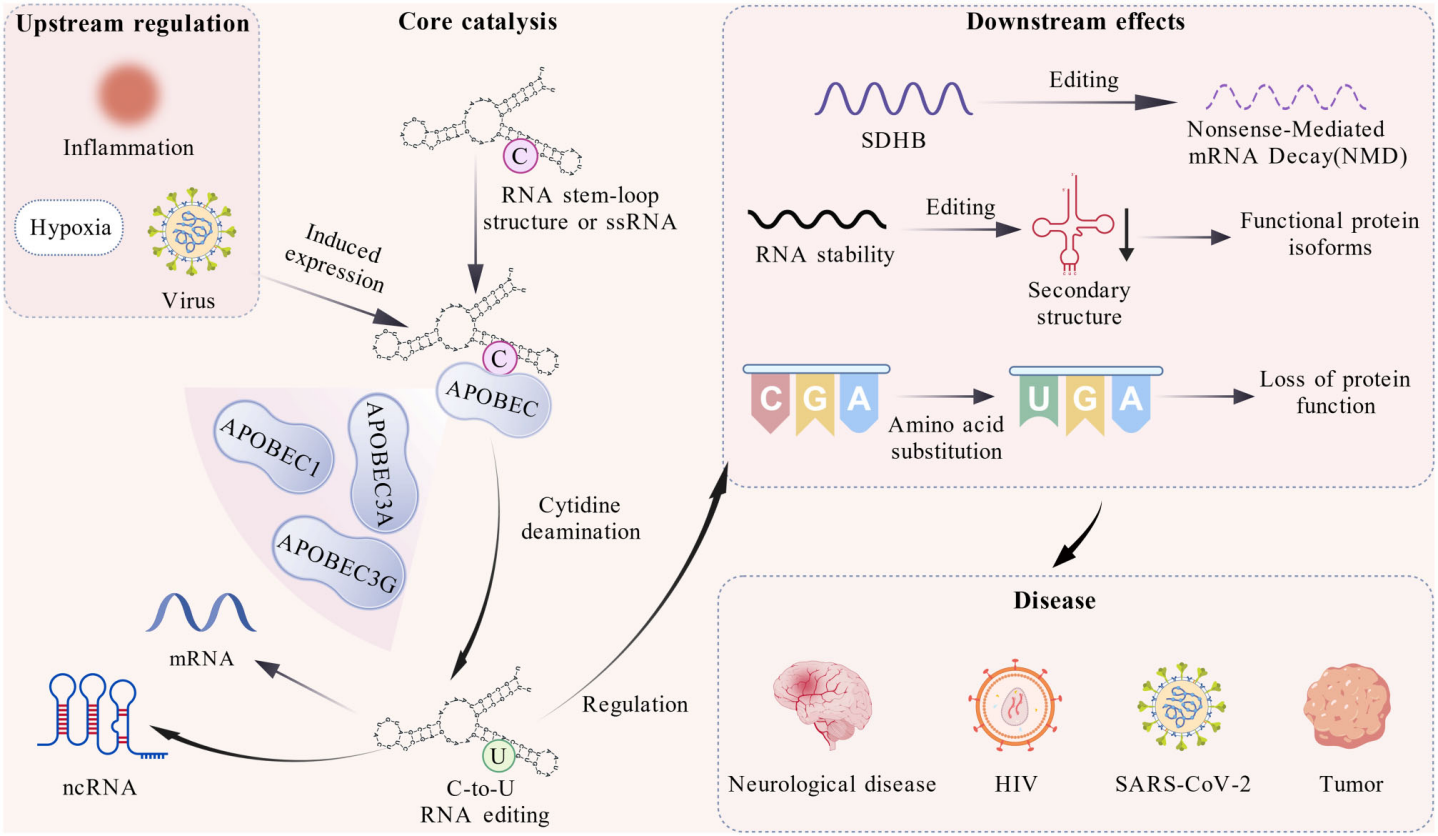
The mechanism of C-to-U RNA editing. The mechanism of cytidine-to-uridine (C-to-U) RNA editing involves upstream regulation, core catalysis, downstream effects, and disease implications. Upstream regulation is influenced by factors like inflammation, hypoxia, and viruses. In core catalysis, APOBEC family members mediate the deamination of cytidine (C) to uridine (U) in RNA stem-loop structures or single-stranded RNA (ssRNA), leading to C-to-U editing in mRNA and non-coding RNA (ncRNA). Downstream effects encompass several aspects: RNA function (e.g. succinate dehydrogenase subunit B (SDHB) editing triggers nonsense-mediated decay (NMD)), RNA stability (generating functional protein isoforms via secondary structure changes), and protein function (amino acid substitution leading to loss of protein function). This mechanism correlates with various diseases, including neurological diseases, HIV, SARS-CoV-2 infection, and tumors.

C-to-U RNA editing represents a post-transcriptional regulatory mechanism whose molecular basis depends on a finely tuned balance between pro-editing and anti-editing factors. This process is essential for maintaining normal physiological function and also contributes significantly to immune regulation and disease development (Fig. 7).

At the molecular level, C-to-U RNA editing is governed by multiple regulatory factors that can be functionally classified as pro–editing cofactors or anti–editing factors. The recently identified RNA binding motif protein 46 (RBM46), a cofactor of APOBEC1, forms an RNA-independent editing complex through its RRM2 and RRM3 domains, significantly enhancing the editing of substrates like apolipoprotein B (apoB) mRNA [438], while APOBEC1 alone induces meaningless mediated decay (NMD) [439]. However, some previously identified factors are dispensable under normal physiological conditions. For instance, the APOBEC1 complementary factor is nonessential for normal C-to-U RNA editing but has a novel role in adult male kidneys [440]. In terms of anti-editing, CUG Binding Protein 2 (CUGBP2), a core component of the apoB mRNA editing holoenzyme, inhibits the editing process in a dose-dependent manner [441].

Besides, C-to-U RNA editing plays a major role in modulating T cell differentiation by altering the targeting of miR-100 from MTOR to SMAD2 [442]. Notably, APOBEC3G induces site-specific C-to-U RNA editing in natural killer cells and lymphoma cell lines under crowded and hypoxic conditions [434].

### The role of C-to-U RNA editing in diseases

C-to-U RNA editing is increasingly recognized for its significant role in human diseases (Table 9). In monocytes and pro-inflammatory macrophages, the cytidine deaminase APOBEC3A induces site-specific C-to-U RNA editing in hundreds of genes under conditions of hypoxia and/or interferon stimulation [436]. These editing sites are notably enriched in genes related to tumors, hypertension, and nervous system diseases, suggesting a potential pathogenic mechanism [436, 443].

**Table 9.** Representative C-to-U RNA editing events in human diseases.

Diseases		Regulators	Functions
Tumors	TGCT	A1CF	Partial A1CF deletion reduces TGCT risk via parental effects, improves testicular atrophy, and reproductive performance [444].
	BC	APOBEC3B	APOBEC3B facilitates BC progression and drug resistance through DNA deamination [445].
Nervous system diseases	iTLE	APOBEC1	APOBEC1 dimorphism as a new genetic risk factor of epilepsy [446].
	HD	hsa-miR-10b-5p	hsa-mir-10b-5p was edited to have an additional cytosine at 5’ end; edited form targets GTPBP10, affecting mitochondrial function and promoting disease progression [447].
Infectious diseases	SARS-CoV-2 infection	Viral S gene	APOBEC mediates C-to-U editing; the S gene’s high editing rate (single-stranded regions preferred) drives viral variation [449].
	HIV-1 infection	Vif	HIV-1 utilizes a viral auxiliary protein known as Vif to degrade the APOBEC3 enzyme in host cells [450].

Abbreviations: TGCT, testicular germ cell tumor; BC, breast cancer; iTLE, intractable temporal lobe epilepsy; HD, Huntington’s disease; SARS-CoV-2, severe acute respiratory syndrome coronavirus 2; HIV-1, human immunodeficiency virus type 1.

During tumorigenesis, the APOBEC1 complementation factor (A1CF) promotes the development of germ cell tumors. Partial deletion of A1CF reduces the risk of testicular germ cell tumor (TGCT) and improves reproductive function [444]. Conversely, APOBEC3B facilitates BC progression and drug resistance primarily through DNA deamination, highlighting the diverse roles of APOBEC family members in tumor biology [445].

In nervous system diseases, polymorphisms in the *APOBEC1* gene are closely associated with the genetic susceptibility to intractable temporal lobe epilepsy (iTLE), indicating a genetic link between editing-related factors and the risk of these diseases [446]. In the prefrontal cortex of Huntington’s disease (HD) patients, specific editing of hsa-miR-10b-5p impacts mitochondrial function by targeting the *GTPBP10* gene, further illustrating how editing processes contribute to the disease’s pathological progression [447].

APOBEC3-mediated C-to-U RNA editing is closely related to the formation of autoantigens. In systemic lupus erythematosus (SLE), this editing process may contribute to the disease’s pathogenesis by enhancing the production of autoantigen [448]. Regarding virus evolution, the C-to-U RNA editing frequency of the SARS-CoV-2 spike protein gene is significantly higher than that of other regions, and the structural characteristics of single-stranded RNA make it more susceptible to APOBEC [449]. Moreover, lentiviruses such as HIV-1 utilize the viral auxiliary protein Vif to degrade the APOBEC3 enzyme in host cells, thus preventing potentially lethal mutations. However, this interaction is not foolproof. A significant level of APOBEC mutations may still occur, potentially promoting immune escape and drug resistance, thereby playing a crucial role in viral adaptation [450].

### C-to-U RNA editing-related targeted therapy

The C-to-U RNA editing site (RES) shows significant potential in developing tumor therapeutic targets. Specific RESs have been found to correlate with multiple clinical features, including overall survival, cancer stemness, and immune cell infiltration. Functional experiments have confirmed that one C-to-U RES in CSNK2B promotes the proliferation of colon cancer cells, while another RES in RPS14 inhibits proliferation, revealing the clinical application prospects of targeting specific C-to-U RESs for personalized cancer treatment [451].

C-to-U RNA editing is a crucial technology that bridges basic research in epitranscriptomics and the clinical application of gene therapy. It holds significant potential for disease treatment and is poised to emerge as an important tool for RNA-level interventions, following the success of mRNA vaccines.

## GlycoRNA

Glycosylated RNA (glycoRNA) is a newly discovered class of modified RNA, which is mainly located on the cell surface [8, 9]. Preliminary functional studies have shown that it is involved in intercellular communication and immune recognition [452]. At the mechanistic level, the specific capture strategy targeting the acp^3^U site demonstrated that endogenous small RNA can inhibit the recognition of TLR3 and TLR7 through N-glycosylation at this site, thereby regulating the fundamental mechanisms of the innate immune response [453, 454]. In addition, glycoRNA, with *N*-Acetylgalactosamine (GalNAc) as a precursor, enters exosomes via the endosomal sorting complexes required for transport (ESCRT) pathway, maintaining stability through coordination with protein glycosylation and facilitating intercellular information transmission [455].

In tumor-related studies, the expression level of glycoRNA on the cell surface is negatively correlated with the degree of malignancy and metastatic potential of tumors [456]. For example, in hepatocellular carcinoma, SERBP1 has been identified as a key regulator of glycoRNA, potentially influencing disease progression through its effects on apoptosis, ribosome function, and taurine metabolism [457]. Additionally, the highly expressed cytoplasmic nucleophosmin 1 (csNPM1) on the surface of AML stem cells is emerging as a potential therapeutic target [458].

From a technical standpoint, the novel dual-recognition FRET strategy allows for precise differentiation of glycoRNAs from various extracellular vesicles, paving the way for advancements in cancer diagnosis [459]. In terms of therapeutic applications, the use of glycoRNA nanoparticles to deliver MT1 siRNA can simultaneously inhibit the formation of neutrophil extracellular traps and improve arterial pathological remodeling in the abdominal aortic aneurysm (AAA) model, showing a promising transformation prospect [460].

As a newly identified type of RNA modification, research on glycoRNA is currently in its early stages. Presently, the focus is primarily on limited areas, such as immune regulation and oncology. Future investigations must undertake a comprehensive analysis of its biological mechanisms, including the modification enzyme system, intracellular transport pathways, and their dynamic regulatory networks in both physiological and pathological contexts. In addition, it is essential to explore its implications in neurological, metabolic, and other diseases, develop high-sensitivity in situ detection technologies, and assess its potential as a novel biomarker or drug target. This will contribute to the establishment of new theoretical frameworks and intervention strategies for disease prevention and treatment.

## Conclusion

RNA modification represents a critical layer of post-transcriptional regulation, establishing a dynamic and reversible regulatory network that is essential for maintaining normal cellular physiology. This regulatory system precisely modulates gene expression at multiple levels, including RNA splicing, nucleoplasmic transport, structural stability, and translation efficiency, through various chemical modifications such as methylation, pseudouridine, and acetylation. These modifications are integral to fundamental biological processes such as cell differentiation, metabolic homeostasis, and immune responses. A finely tuned regulatory mechanism is achieved through the synergistic actions of writer, eraser, and reader proteins, enabling cells to maintain proper function in complex environments [461, 462]. Notably, extensive interactions exist among different types of RNA modifications and are influenced by environmental factors.

Disruption of the RNA modification network can lead to the onset and progression of various diseases. In cancer, aberrant expression of m^6^A-modifying enzymes such as METTL3 and FTO drives malignant phenotypes, including cell proliferation, metastasis, metabolic reprogramming, and immune evasion [463]. Concurrently, DKC1-mediated Ψ modification and NAT10-catalysed ac^4^C modification further promote tumor progression by influencing rRNA function and enhancing mRNA stability, respectively [464, 465]. Besides, ADAR1-mediated A-to-I editing plays a critical role in maintaining immune homeostasis through the modification of immunogenic double-stranded RNA, and its overexpression in various tumors is closely associated with malignant characteristics [466, 467].

Given the critical regulatory role of RNA modifications, their associated proteins have emerged as potential therapeutic targets and biomarkers. The modulation of YTHDF1 can reinstate the anti-tumor immune response [468], while targeting ADAR1 offers a novel approach for cancer treatment. It is important to note that although numerous RNA modification-related therapeutic targets have been identified in multiple diseases, research on their combinatorial application in pathological states remains limited.

In summary, RNA modification serves as a multi-level and dynamically adjustable molecular regulatory network, providing a fundamental basis for understanding the mechanisms of biological processes and offering significant breakthroughs in exploring novel disease treatment strategies.
